# [μ-Bis(diphenyl­arsino)methane-1:2κ^2^ *As*:*As*’]nona­carbonyl-1κ^3^ *C*,2κ^3^ *C*,3κ^3^ *C*-(triphenyl phosphite-3κ*P*)-*triangulo*-triruthenium(0)

**DOI:** 10.1107/S1600536810001200

**Published:** 2010-01-30

**Authors:** Omar bin Shawkataly, Imthyaz Ahmed Khan, Chin Sing Yeap, Hoong-Kun Fun

**Affiliations:** a Chemical Sciences Programme, School of Distance Education, Universiti Sains Malaysia, 11800 USM, Penang, Malaysia; b X-ray Crystallography Unit, School of Physics, Universiti Sains Malaysia, 11800 USM, Penang, Malaysia

## Abstract

The asymmetric unit of the title *triangulo*-triruthenium compound, [Ru_3_(C_25_H_22_As_2_)(C_18_H_15_O_3_P)(CO)_9_], contains two crystallographically independent but similar mol­ecules. The bis­(diphenyl­arsino)methane ligand bridges an Ru—Ru bond and the monodentate phosphite ligand bonds to the third Ru atom. Both the phosphite and arsine ligands are equatorial with respect to the Ru_3_ triangle. In addition, each Ru atom carries one equatorial and two axial terminal carbonyl ligands. One of the triphenyl­phosphite benzene rings in one of the mol­ecules is disordered over two positions with refined site occupancies of 0.60 (3) and 0.40 (3). In the crystal packing, the mol­ecules are stacked along *a* axis. Intra­molecular C—H⋯O hydrogen bonds stabilize the mol­ecular structure and weak inter­molecular C—H⋯π inter­actions further stabilize the crystal structure. The crystal studied was a non-merohedral twin, the refined ratio of the twin components being 0.618 (1):0.382 (1).

## Related literature

For general background to *triangulo*-triruthenium derivatives, see: Bruce *et al.* (1985 ▶, 1988*a* ▶,*b* ▶). For related structures, see: Shawkataly *et al.* (1998 ▶, 2004 ▶, 2009 ▶). For the synthesis of μ-bis­(diphenyl­arsino)methane­deca­carbonyl­triruthenium(0), see: Bruce *et al.* (1983 ▶). For stability of the temperature controller used for the data collection, see: Cosier & Glazer (1986 ▶).
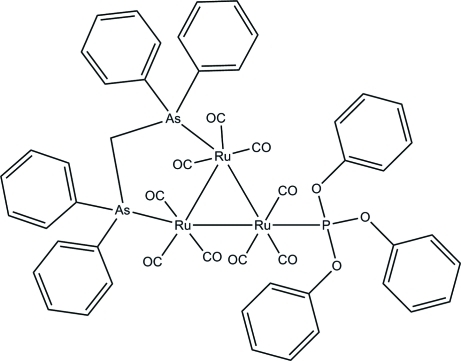


## Experimental

### 

#### Crystal data

[Ru_3_(C_25_H_22_As_2_)(C_18_H_15_O_3_P)(CO)_9_]
*M* *_r_* = 1337.84
Monoclinic, 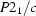

*a* = 15.6537 (3) Å
*b* = 21.3662 (4) Å
*c* = 30.1698 (6) Å
β = 90.125 (1)°
*V* = 10090.6 (3) Å^3^
*Z* = 8
Mo *K*α radiation
μ = 2.28 mm^−1^
*T* = 100 K
0.25 × 0.23 × 0.04 mm

#### Data collection

Bruker SMART APEXII CCD area-detector diffractometer
Absorption correction: multi-scan (*SADABS*; Bruker, 2005 ▶) *T*_min_ = 0.604, *T*_max_ = 0.918
235438 measured reflections
29385 independent reflections
22171 reflections with *I* > 2σ(*I*)
*R*_int_ = 0.080

#### Refinement

*R*[*F* ^2^ > 2σ(*F* ^2^)] = 0.059
*wR*(*F* ^2^) = 0.132
*S* = 1.07
29385 reflections
1275 parameters
174 restraints
H-atom parameters constrained
Δρ_max_ = 2.75 e Å^−3^
Δρ_min_ = −2.02 e Å^−3^

### 

Data collection: *APEX2* (Bruker, 2005 ▶); cell refinement: *SAINT* (Bruker, 2005 ▶); data reduction: *SAINT*; program(s) used to solve structure: *SHELXTL* (Sheldrick, 2008 ▶); program(s) used to refine structure: *SHELXTL*; molecular graphics: *SHELXTL*; software used to prepare material for publication: *SHELXTL* and *PLATON* (Spek, 2009 ▶).

## Supplementary Material

Crystal structure: contains datablocks global, I. DOI: 10.1107/S1600536810001200/tk2607sup1.cif

Structure factors: contains datablocks I. DOI: 10.1107/S1600536810001200/tk2607Isup2.hkl

Additional supplementary materials:  crystallographic information; 3D view; checkCIF report

## Figures and Tables

**Table 1:** Hydrogen-bond geometry (Å, °) *Cg*1, *Cg*2, *Cg*3, *Cg*4, *Cg*5, *Cg*6 and *Cg*7 are the centroids of the C1*B*–C6*B*, C1*A*–C6*A*, C14*A*–C19*A*, C14*A*–C19*A*, C7*A*–C12*A*, C20*B*–C25*B* and C20*A*–C25*A* benzene rings, respectively.

*D*—H⋯*A*	*D*—H	H⋯*A*	*D*⋯*A*	*D*—H⋯*A*
C31*A*—H31*A*⋯O12*A*	0.93	2.42	3.014 (13)	121
C39*A*—H39*A*⋯O11*A*	0.93	2.38	2.959 (11)	120
C43*B*—H43*B*⋯O2*B*	0.93	2.53	3.42 (2)	158
C11*A*—H11*A*⋯*Cg*1^i^	0.93	2.95	3.742 (12)	144
C11*B*—H11*B*⋯*Cg*2^ii^	0.93	2.85	3.664 (10)	147
C16*A*—H16*A*⋯*Cg*1^iii^	0.93	2.82	3.668 (10)	151
C16*B*—H16*B*⋯*Cg*2	0.93	2.82	3.627 (10)	146
C24*A*—H24*A*⋯*Cg*3^iv^	0.93	2.96	3.719 (10)	140
C24*B*—H24*B*⋯*Cg*4^v^	0.93	2.91	3.679 (10)	141
C36*B*—H36*B*⋯*Cg*5^ii^	0.93	2.85	3.756 (12)	166
C40*A*—H40*A*⋯*Cg*6^vi^	0.93	2.82	3.660 (10)	150
C40*B*—H40*B*⋯*Cg*7^vii^	0.93	2.71	3.55 (2)	150
C40*C*—H40*C*⋯*Cg*7^vii^	0.93	2.70	3.56 (3)	154

Symmetry codes: (i) 
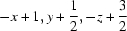
; (ii) 
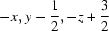
; (iii) 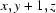; (iv) 
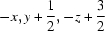
; (v) 
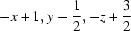
; (vi) 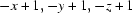; (vii) 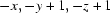.

**Fig. 1.**
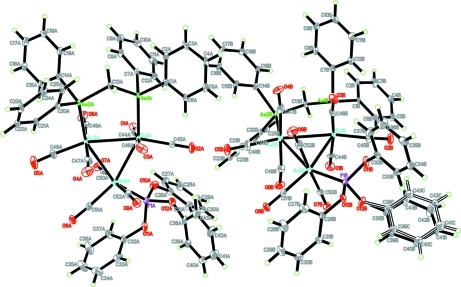
The molecular structure of the title compound with 30% probability ellipsoids for non-H atoms. All disordered components are shown.

**Fig. 2.**
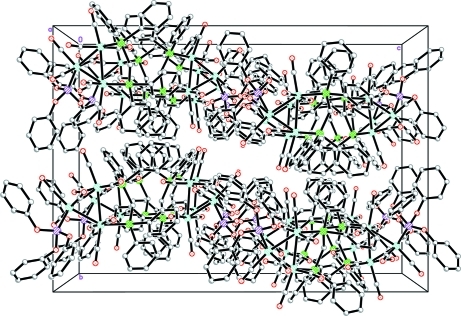
The crystal packing of the title compound, viewed down the a axis, showing the molecules stacked along a axis. Hydrogen atoms not involved in the hydrogen-bonding (dashed lines) have been omitted for clarity. Only major disordered component is shown.

## supplementary crystallographic information

### Comment

*Triangulo*-triruthenium clusters are known for their interesting structural variations and related catalytic activity. A large number of substituted derivatives, Ru_3_(CO)_12-n_*L*_n_ (*L* = group 15 ligand) have been reported (Bruce, Liddell *et al.*, 1988*a*, b; Bruce *et al.*, 1985). As part of our study on the substitution of transition metal-carbonyl clusters with mixed-ligand complexes, we have published several structures of *triangulo*-triruthenium-carbonyl clusters containing mixed P/As and P/Sb ligands (Shawkataly *et al.*, 1998, 2004, 2009). Herein, we report the synthesis and structure of Ru_3_(C_18_H_15_O_3_P)(C_25_H_22_As_2_)(CO)_9_.

The asymmetric unit consists of two crystallographically independent molecules of the *triangulo*-triruthenium complex, *A* and *B* (Fig. 1). The bond lengths and angles of title compound are comparable to those found in a closely related structure (Shawkataly *et al.*, 2009). The bis(diphenylarsino)methane ligand bridges the Ru1—Ru2 bond and the monodentate phosphine ligand bonds to the Ru3 atom. Both the phosphine and arsine ligands are equatorial with respect to the Ru_3_ triangle. Additionally, each Ru atom carries one equatorial and two axial terminal carbonyl ligands. The triphenylphosphite benzene rings make dihedral angles (C26–C31/C32–C37, C26–C31/C38–C43 and C32–C37/C38–C43) of 66.9 (5), 55.6 (5) and 62.0 (5)° with each other in molecule *A* whereas these angles are 85.5 (5), 71.4 (9) and 85.8 (9)° (major component), 85.5 (5), 71.2 (11) and 85.1 (11)° (minor component) in molecule *B*. The dihedral angles between the two benzene rings (C1–C6/C7–C12 and C14–C19/C20–C25) are 84.7 (4) and 84.0 (4)° for the two diphenylarsino groups in molecule *A* whereas these angles are 85.0 (4) and 86.3 (4)° in molecule *B*. In the crystal structure, the molecules are stacked along *a* axis (Fig. 2). Intramolecular C31A—H31A···O11A, C39A—H39A···O12A and C43B—H43B···O2B hydrogen bonds stabilize the molecular structure and weak intermolecular C—H···π interactions further stabilize the crystal structure (Table 1).

### Experimental

All manipulations were performed under a dry oxygen-free dinitrogen atmosphere using standard Schlenk techniques, and all solvents were dried over sodium and distilled from sodium benzophenone ketyl under nitrogen. Triphenylphosphite (BDH) was used as received and µ-bis(diphenylarsino)methanedecacarbonyltriruthenium(0) was prepared by a reported procedure (Bruce *et al.*, 1983). The title compound was obtained by refluxing equimolar quantities of Ru_3_(CO)_10_(µ-Ph_2_AsCH_2_AsPh_2_) (105.5 mg, 0.1 mmol) and triphenylphosphite (31.0 mg, 0.1 mmol) in hexane under nitrogen atmosphere. Crystals were grown by slow solvent / solvent diffusion of CH_3_OH into CH_2_Cl_2_.

### Refinement

The C38B–C43B benzene ring is disordered over two positions with refined site occupancies of 0.60 (3) and 0.40 (3). The minor component of the disordered atoms was refined isotropically. The same U_ij_ parameters were used for the atom pairs O2A/O2B and O11A/O11B. All disordered atoms were subjected to rigid bond and similarity restraints. All hydrogen atoms were positioned geometrically and refined using a riding model with C—H = 0.93–0.97 Å and *U*_iso_(H) = 1.2 *U*_eq_(C). The maximum and minimum residual electron density peaks of 2.75 and -2.02 e Å^-3^, respectively, were located 1.52 Å and 0.05 Å from the Ru1A and Ru2B atoms, respectively. The crystal studied is a twin (twin law: -1 0 0/0 - 1 0/0 0 1) with the refined ratio of twin components being 0.618 (1):0.382 (1).

### Crystal data

**Table:**

[Ru_3_(C_25_H_22_As_2_)(C_18_H_15_O_3_P)(CO)_9_]	*F*(000) = 5264
*M**_r_* = 1337.84	*D*_x_ = 1.761 Mg m^−^^3^
Monoclinic, *P*2_1_/*c*	Mo *K*α radiation, λ = 0.71073 Å
Hall symbol: -P 2ybc	Cell parameters from 9830 reflections
*a* = 15.6537 (3) Å	θ = 2.4–29.2°
*b* = 21.3662 (4) Å	µ = 2.28 mm^−^^1^
*c* = 30.1698 (6) Å	*T* = 100 K
β = 90.125 (1)°	Block, red
*V* = 10090.6 (3) Å^3^	0.25 × 0.23 × 0.04 mm
*Z* = 8	

### Data collection

**Table:**

Bruker SMART APEXII CCD area-detector diffractometer	29385 independent reflections
Radiation source: fine-focus sealed tube	22171 reflections with *I* > 2σ(*I*)
graphite	*R*_int_ = 0.080
φ and ω scans	θ_max_ = 30.0°, θ_min_ = 1.0°
Absorption correction: multi-scan (*SADABS*; Bruker, 2005)	*h* = −21→21
*T*_min_ = 0.604, *T*_max_ = 0.918	*k* = −30→29
235438 measured reflections	*l* = −41→42

### Refinement

**Table:**

Refinement on *F*^2^	Primary atom site location: structure-invariant direct methods
Least-squares matrix: full	Secondary atom site location: difference Fourier map
*R*[*F*^2^ > 2σ(*F*^2^)] = 0.059	Hydrogen site location: inferred from neighbouring sites
*wR*(*F*^2^) = 0.132	H-atom parameters constrained
*S* = 1.07	*w* = 1/[σ^2^(*F*_o_^2^) + (0.0243*P*)^2^ + 105.7247*P*] where *P* = (*F*_o_^2^ + 2*F*_c_^2^)/3
29385 reflections	(Δ/σ)_max_ = 0.001
1275 parameters	Δρ_max_ = 2.75 e Å^−^^3^
174 restraints	Δρ_min_ = −2.02 e Å^−^^3^

### Special details

**Table:**

Experimental. The crystal was placed in the cold stream of an Oxford Cyrosystems Cobra open-flow nitrogen cryostat (Cosier & Glazer, 1986) operating at 100.0 (1) K.
Geometry. All e.s.d.'s (except the e.s.d. in the dihedral angle between two l.s. planes) are estimated using the full covariance matrix. The cell e.s.d.'s are taken into account individually in the estimation of e.s.d.'s in distances, angles and torsion angles; correlations between e.s.d.'s in cell parameters are only used when they are defined by crystal symmetry. An approximate (isotropic) treatment of cell e.s.d.'s is used for estimating e.s.d.'s involving l.s. planes.
Refinement. Refinement of *F*^2^ against ALL reflections. The weighted *R*-factor *wR* and goodness of fit *S* are based on *F*^2^, conventional *R*-factors *R* are based on *F*, with *F* set to zero for negative *F*^2^. The threshold expression of *F*^2^ > σ(*F*^2^) is used only for calculating *R*-factors(gt) *etc*. and is not relevant to the choice of reflections for refinement. *R*-factors based on *F*^2^ are statistically about twice as large as those based on *F*, and *R*- factors based on ALL data will be even larger.

### Fractional atomic coordinates and isotropic or equivalent isotropic displacement parameters (Å^2^)

**Table:**

	*x*	*y*	*z*	*U*_iso_*/*U*_eq_	Occ. (<1)
Ru1A	0.28173 (4)	0.76421 (3)	0.64426 (2)	0.01788 (13)	
Ru2A	0.26063 (4)	0.89647 (3)	0.63474 (2)	0.01799 (13)	
Ru3A	0.35416 (4)	0.82713 (3)	0.57030 (2)	0.01912 (13)	
As1A	0.20409 (5)	0.76330 (3)	0.71392 (3)	0.01517 (16)	
As2A	0.15232 (6)	0.90735 (3)	0.69237 (3)	0.01558 (16)	
P1A	0.43840 (15)	0.74851 (10)	0.54556 (8)	0.0236 (5)	
O1A	0.4568 (4)	0.7873 (3)	0.6862 (3)	0.0389 (18)	
O2A	0.3396 (5)	0.6296 (3)	0.6344 (3)	0.0470 (13)	
O3A	0.1042 (5)	0.7454 (4)	0.6046 (3)	0.045 (2)	
O4A	0.1215 (4)	0.8772 (4)	0.5657 (3)	0.046 (2)	
O5A	0.2849 (5)	1.0281 (3)	0.5992 (3)	0.054 (2)	
O6A	0.4037 (5)	0.9200 (4)	0.7014 (3)	0.0425 (19)	
O7A	0.5087 (5)	0.8852 (4)	0.6168 (3)	0.0369 (17)	
O8A	0.3598 (5)	0.9325 (3)	0.5021 (2)	0.0327 (16)	
O9A	0.2098 (4)	0.7605 (3)	0.5196 (2)	0.0336 (15)	
O10A	0.5103 (4)	0.7324 (3)	0.5814 (2)	0.0398 (16)	
O11A	0.4931 (4)	0.7551 (3)	0.5012 (2)	0.0294 (10)	
O12A	0.4009 (5)	0.6802 (3)	0.5398 (2)	0.0373 (16)	
C1A	0.1324 (5)	0.6910 (3)	0.7279 (3)	0.0186 (17)	
C2A	0.0884 (6)	0.6898 (4)	0.7683 (3)	0.0215 (18)	
H2AA	0.0953	0.7224	0.7884	0.026*	
C3A	0.0346 (6)	0.6401 (4)	0.7786 (3)	0.0237 (19)	
H3AA	0.0046	0.6400	0.8052	0.028*	
C4A	0.0258 (6)	0.5904 (4)	0.7490 (3)	0.028 (2)	
H4AA	−0.0089	0.5565	0.7560	0.033*	
C5A	0.0693 (6)	0.5918 (4)	0.7088 (3)	0.0244 (19)	
H5AA	0.0622	0.5593	0.6887	0.029*	
C6A	0.1239 (6)	0.6419 (3)	0.6983 (3)	0.0223 (18)	
H6AA	0.1539	0.6420	0.6717	0.027*	
C7A	0.2706 (6)	0.7713 (3)	0.7674 (3)	0.0207 (17)	
C8A	0.2528 (6)	0.8145 (4)	0.8010 (3)	0.0262 (19)	
H8AA	0.2052	0.8403	0.7988	0.031*	
C9A	0.3057 (7)	0.8186 (4)	0.8371 (3)	0.031 (2)	
H9AA	0.2943	0.8478	0.8591	0.038*	
C10A	0.3754 (7)	0.7801 (5)	0.8411 (3)	0.034 (2)	
H10A	0.4107	0.7831	0.8659	0.041*	
C11A	0.3934 (7)	0.7359 (5)	0.8079 (4)	0.035 (2)	
H11A	0.4405	0.7097	0.8106	0.042*	
C12A	0.3411 (6)	0.7316 (4)	0.7713 (3)	0.0266 (19)	
H12A	0.3527	0.7023	0.7493	0.032*	
C13A	0.1143 (5)	0.8278 (3)	0.7180 (3)	0.0164 (16)	
H13A	0.0636	0.8134	0.7025	0.020*	
H13B	0.0994	0.8341	0.7489	0.020*	
C14A	0.1714 (5)	0.9620 (4)	0.7431 (3)	0.0193 (17)	
C15A	0.2239 (6)	1.0135 (3)	0.7376 (3)	0.0211 (17)	
H15A	0.2541	1.0185	0.7114	0.025*	
C16A	0.2318 (6)	1.0574 (4)	0.7709 (3)	0.0267 (19)	
H16A	0.2679	1.0916	0.7672	0.032*	
C17A	0.1858 (6)	1.0506 (4)	0.8101 (4)	0.030 (2)	
H17A	0.1909	1.0804	0.8324	0.036*	
C18A	0.1323 (6)	0.9995 (4)	0.8159 (3)	0.0240 (18)	
H18A	0.1016	0.9949	0.8420	0.029*	
C19A	0.1251 (5)	0.9556 (4)	0.7824 (3)	0.0216 (18)	
H19A	0.0891	0.9214	0.7862	0.026*	
C20A	0.0468 (6)	0.9467 (4)	0.6727 (3)	0.0184 (16)	
C21A	0.0568 (5)	0.9991 (3)	0.6452 (3)	0.0183 (15)	
H21A	0.1107	1.0107	0.6352	0.022*	
C22A	−0.0142 (6)	1.0331 (4)	0.6330 (3)	0.0240 (18)	
H22A	−0.0079	1.0681	0.6149	0.029*	
C23A	−0.0957 (6)	1.0160 (4)	0.6475 (3)	0.027 (2)	
H23A	−0.1434	1.0389	0.6389	0.033*	
C24A	−0.1044 (6)	0.9639 (4)	0.6752 (3)	0.026 (2)	
H24A	−0.1582	0.9522	0.6853	0.032*	
C25A	−0.0323 (5)	0.9291 (4)	0.6878 (3)	0.0213 (17)	
H25A	−0.0381	0.8944	0.7061	0.026*	
C26A	0.5823 (6)	0.6930 (4)	0.5772 (4)	0.032 (2)	
C27A	0.6492 (8)	0.7085 (5)	0.6032 (4)	0.043 (3)	
H27A	0.6465	0.7435	0.6215	0.052*	
C28A	0.7219 (7)	0.6715 (5)	0.6025 (4)	0.045 (3)	
H28A	0.7686	0.6818	0.6202	0.054*	
C29A	0.7251 (6)	0.6196 (4)	0.5756 (4)	0.039 (2)	
H29A	0.7735	0.5944	0.5757	0.047*	
C30A	0.6594 (6)	0.6053 (4)	0.5494 (3)	0.032 (2)	
H30A	0.6634	0.5706	0.5309	0.038*	
C31A	0.5850 (7)	0.6409 (5)	0.5491 (4)	0.035 (2)	
H31A	0.5389	0.6304	0.5310	0.042*	
C32A	0.5065 (6)	0.8110 (4)	0.4778 (4)	0.029 (2)	
C33A	0.4591 (7)	0.8208 (5)	0.4406 (4)	0.037 (2)	
H33A	0.4180	0.7919	0.4318	0.045*	
C34A	0.4733 (7)	0.8752 (6)	0.4157 (4)	0.051 (3)	
H34A	0.4425	0.8819	0.3898	0.061*	
C35A	0.5316 (7)	0.9181 (6)	0.4292 (5)	0.053 (3)	
H35A	0.5394	0.9547	0.4130	0.063*	
C36A	0.5788 (8)	0.9075 (6)	0.4666 (5)	0.058 (4)	
H36A	0.6200	0.9364	0.4752	0.070*	
C37A	0.5656 (7)	0.8529 (5)	0.4922 (4)	0.038 (2)	
H37A	0.5963	0.8460	0.5182	0.046*	
C38A	0.3726 (6)	0.6460 (4)	0.5032 (4)	0.026 (2)	
C39A	0.4202 (6)	0.6384 (4)	0.4643 (3)	0.026 (2)	
H39A	0.4707	0.6608	0.4607	0.032*	
C40A	0.3936 (7)	0.5991 (4)	0.4321 (3)	0.039 (2)	
H40A	0.4270	0.5924	0.4071	0.046*	
C41A	0.3145 (7)	0.5687 (5)	0.4370 (4)	0.045 (3)	
H41A	0.2950	0.5426	0.4145	0.054*	
C42A	0.2655 (6)	0.5764 (4)	0.4743 (4)	0.040 (2)	
H42A	0.2126	0.5570	0.4768	0.048*	
C43A	0.2973 (7)	0.6145 (4)	0.5089 (4)	0.042 (2)	
H43A	0.2672	0.6182	0.5354	0.051*	
C44A	0.3913 (6)	0.7820 (4)	0.6693 (3)	0.027 (2)	
C45A	0.3155 (7)	0.6793 (4)	0.6376 (3)	0.029 (2)	
C46A	0.1712 (6)	0.7545 (4)	0.6167 (3)	0.029 (2)	
C47A	0.1757 (6)	0.8804 (4)	0.5915 (3)	0.029 (2)	
C48A	0.2756 (7)	0.9794 (4)	0.6137 (4)	0.036 (2)	
C49A	0.3526 (7)	0.9068 (4)	0.6772 (3)	0.029 (2)	
C50A	0.4490 (6)	0.8638 (4)	0.6023 (3)	0.0236 (19)	
C51A	0.3595 (6)	0.8920 (4)	0.5265 (3)	0.0260 (19)	
C52A	0.2589 (5)	0.7859 (4)	0.5402 (3)	0.0235 (17)	
Ru1B	0.22582 (4)	0.27062 (3)	0.64659 (2)	0.01799 (13)	
Ru2B	0.24102 (4)	0.40383 (3)	0.64090 (2)	0.01776 (13)	
Ru3B	0.15314 (4)	0.33614 (3)	0.57451 (2)	0.01808 (13)	
As1B	0.29672 (5)	0.26613 (3)	0.71723 (3)	0.01568 (16)	
As2B	0.34802 (6)	0.41124 (3)	0.69863 (3)	0.01577 (16)	
P1B	0.08199 (14)	0.25486 (9)	0.54490 (8)	0.0205 (4)	
O1B	0.4092 (4)	0.2680 (3)	0.6091 (3)	0.0393 (18)	
O2B	0.1823 (5)	0.1329 (3)	0.6334 (3)	0.0470 (13)	
O3B	0.0452 (5)	0.2821 (3)	0.6864 (3)	0.0347 (17)	
O4B	0.0959 (5)	0.4135 (4)	0.7089 (3)	0.0399 (18)	
O5B	0.2015 (5)	0.5377 (3)	0.6150 (3)	0.0427 (19)	
O6B	0.3839 (5)	0.4010 (4)	0.5702 (3)	0.0411 (18)	
O7B	0.3105 (4)	0.2841 (3)	0.5243 (2)	0.0306 (14)	
O8B	0.1389 (5)	0.4418 (3)	0.5070 (2)	0.0333 (16)	
O9B	−0.0092 (5)	0.3839 (4)	0.6217 (2)	0.0375 (18)	
O10B	0.0052 (4)	0.2665 (3)	0.5104 (2)	0.0238 (13)	
O11B	0.0345 (4)	0.2080 (2)	0.5758 (2)	0.0294 (10)	
O12B	0.1469 (4)	0.2095 (3)	0.5183 (2)	0.0292 (13)	
C1B	0.3687 (6)	0.1935 (3)	0.7305 (3)	0.0185 (17)	
C2B	0.4093 (6)	0.1907 (4)	0.7702 (3)	0.0232 (19)	
H2BA	0.4001	0.2217	0.7913	0.028*	
C3B	0.4653 (6)	0.1410 (4)	0.7794 (4)	0.030 (2)	
H3BA	0.4929	0.1387	0.8066	0.037*	
C4B	0.4792 (6)	0.0956 (4)	0.7477 (3)	0.029 (2)	
H4BA	0.5168	0.0629	0.7535	0.035*	
C5B	0.4377 (7)	0.0984 (4)	0.7077 (4)	0.030 (2)	
H5BA	0.4462	0.0670	0.6868	0.036*	
C6B	0.3832 (6)	0.1479 (4)	0.6981 (3)	0.0239 (19)	
H6BA	0.3567	0.1507	0.6705	0.029*	
C7B	0.2250 (6)	0.2699 (4)	0.7697 (3)	0.0205 (17)	
C8B	0.2363 (6)	0.3124 (4)	0.8044 (3)	0.0252 (19)	
H8BA	0.2813	0.3408	0.8036	0.030*	
C9B	0.1797 (7)	0.3123 (4)	0.8405 (3)	0.031 (2)	
H9BA	0.1879	0.3403	0.8637	0.037*	
C10B	0.1118 (6)	0.2707 (4)	0.8417 (4)	0.030 (2)	
H10B	0.0738	0.2713	0.8653	0.036*	
C11B	0.1009 (6)	0.2289 (4)	0.8079 (3)	0.030 (2)	
H11B	0.0561	0.2003	0.8088	0.035*	
C12B	0.1564 (6)	0.2289 (4)	0.7723 (3)	0.0221 (17)	
H12B	0.1476	0.2005	0.7494	0.027*	
C13B	0.3844 (5)	0.3313 (3)	0.7240 (3)	0.0196 (17)	
H13C	0.3969	0.3370	0.7552	0.023*	
H13D	0.4365	0.3177	0.7095	0.023*	
C14B	0.3287 (5)	0.4648 (4)	0.7504 (3)	0.0201 (17)	
C15B	0.2751 (6)	0.5159 (4)	0.7457 (3)	0.0233 (18)	
H15B	0.2434	0.5204	0.7198	0.028*	
C16B	0.2678 (6)	0.5603 (4)	0.7789 (3)	0.028 (2)	
H16B	0.2334	0.5953	0.7746	0.034*	
C17B	0.3106 (6)	0.5527 (4)	0.8177 (3)	0.0244 (19)	
H17B	0.3043	0.5818	0.8404	0.029*	
C19B	0.3742 (5)	0.4573 (4)	0.7893 (3)	0.0210 (17)	
H19B	0.4110	0.4235	0.7928	0.025*	
C18B	0.3644 (6)	0.5010 (4)	0.8234 (3)	0.0241 (18)	
H18B	0.3938	0.4957	0.8500	0.029*	
C20B	0.4551 (6)	0.4482 (3)	0.6796 (3)	0.0195 (17)	
C21B	0.4517 (6)	0.4984 (4)	0.6507 (3)	0.0249 (19)	
H21B	0.3990	0.5107	0.6394	0.030*	
C22B	0.5240 (7)	0.5309 (4)	0.6380 (3)	0.029 (2)	
H22B	0.5203	0.5641	0.6182	0.034*	
C23B	0.6013 (7)	0.5131 (4)	0.6552 (3)	0.032 (2)	
H23B	0.6504	0.5348	0.6474	0.038*	
C24B	0.6068 (6)	0.4637 (4)	0.6839 (3)	0.028 (2)	
H24B	0.6597	0.4520	0.6952	0.033*	
C25B	0.5344 (6)	0.4309 (4)	0.6962 (3)	0.0248 (19)	
H25B	0.5390	0.3972	0.7155	0.030*	
C26B	0.0026 (6)	0.3163 (4)	0.4807 (3)	0.0232 (18)	
C27B	−0.0607 (6)	0.3607 (5)	0.4872 (4)	0.032 (2)	
H27B	−0.0971	0.3580	0.5114	0.039*	
C28B	−0.0686 (7)	0.4087 (5)	0.4572 (4)	0.041 (3)	
H28B	−0.1108	0.4389	0.4610	0.049*	
C29B	−0.0129 (7)	0.4122 (4)	0.4211 (4)	0.035 (2)	
H29B	−0.0187	0.4447	0.4008	0.042*	
C30B	0.0487 (6)	0.3693 (4)	0.4153 (3)	0.030 (2)	
H30B	0.0852	0.3724	0.3911	0.036*	
C31B	0.0583 (6)	0.3208 (4)	0.4448 (3)	0.0253 (19)	
H31B	0.1014	0.2913	0.4409	0.030*	
C32B	−0.0539 (6)	0.1892 (4)	0.5767 (3)	0.0238 (18)	
C33B	−0.0819 (6)	0.1475 (4)	0.5453 (3)	0.0253 (19)	
H33B	−0.0452	0.1335	0.5232	0.030*	
C34B	−0.1656 (6)	0.1267 (4)	0.5470 (3)	0.030 (2)	
H34B	−0.1854	0.0989	0.5256	0.036*	
C35B	−0.2202 (6)	0.1468 (4)	0.5802 (3)	0.032 (2)	
H35B	−0.2761	0.1322	0.5813	0.038*	
C36B	−0.1908 (7)	0.1891 (5)	0.6119 (4)	0.041 (3)	
H36B	−0.2264	0.2029	0.6345	0.049*	
C37B	−0.1064 (6)	0.2102 (4)	0.6089 (4)	0.034 (2)	
H37B	−0.0862	0.2392	0.6294	0.041*	
C38B	0.1337 (15)	0.1524 (7)	0.4998 (7)	0.024 (4)	0.60 (3)
C39B	0.0950 (13)	0.1458 (6)	0.4591 (6)	0.021 (3)	0.60 (3)
H39B	0.0793	0.1808	0.4427	0.025*	0.60 (3)
C40B	0.080 (2)	0.0872 (8)	0.4431 (7)	0.035 (6)	0.60 (3)
H40B	0.0516	0.0827	0.4161	0.042*	0.60 (3)
C41B	0.1051 (17)	0.0348 (6)	0.4656 (6)	0.040 (4)	0.60 (3)
H41B	0.0891	−0.0049	0.4560	0.048*	0.60 (3)
C42B	0.156 (2)	0.0426 (8)	0.5042 (7)	0.060 (6)	0.60 (3)
H42B	0.1864	0.0091	0.5162	0.071*	0.60 (3)
C43B	0.1608 (17)	0.1005 (7)	0.5231 (7)	0.042 (5)	0.60 (3)
H43B	0.1820	0.1050	0.5518	0.050*	0.60 (3)
C38C	0.117 (2)	0.1477 (10)	0.5043 (9)	0.018 (6)*	0.40 (3)
C39C	0.074 (2)	0.1428 (11)	0.4646 (9)	0.021 (6)*	0.40 (3)
H39C	0.0507	0.1780	0.4510	0.025*	0.40 (3)
C40C	0.065 (3)	0.0850 (12)	0.4458 (10)	0.024 (6)*	0.40 (3)
H40C	0.0560	0.0817	0.4155	0.029*	0.40 (3)
C41C	0.0708 (19)	0.0318 (11)	0.4710 (8)	0.036 (6)*	0.40 (3)
H41C	0.0494	−0.0063	0.4609	0.043*	0.40 (3)
C42C	0.1105 (16)	0.0373 (9)	0.5131 (7)	0.022 (5)*	0.40 (3)
H42C	0.1238	0.0015	0.5293	0.026*	0.40 (3)
C43C	0.1294 (19)	0.0950 (10)	0.5299 (8)	0.029 (6)*	0.40 (3)
H43C	0.1503	0.0988	0.5587	0.035*	0.40 (3)
C44B	0.3398 (6)	0.2701 (4)	0.6208 (3)	0.0255 (19)	
C45B	0.1993 (6)	0.1853 (4)	0.6371 (3)	0.028 (2)	
C46B	0.1126 (6)	0.2808 (4)	0.6710 (3)	0.026 (2)	
C47B	0.1497 (7)	0.4062 (4)	0.6822 (4)	0.031 (2)	
C48B	0.2187 (7)	0.4875 (4)	0.6247 (4)	0.036 (2)	
C49B	0.3283 (6)	0.3990 (4)	0.5953 (3)	0.027 (2)	
C50B	0.2552 (6)	0.3038 (4)	0.5450 (3)	0.0236 (17)	
C51B	0.1415 (6)	0.4010 (4)	0.5322 (3)	0.0252 (19)	
C52B	0.0540 (7)	0.3662 (4)	0.6067 (3)	0.029 (2)	

### Atomic displacement parameters (Å^2^)

**Table:**

	*U*^11^	*U*^22^	*U*^33^	*U*^12^	*U*^13^	*U*^23^
Ru1A	0.0197 (3)	0.0122 (2)	0.0218 (3)	−0.0001 (2)	0.0014 (3)	0.0005 (2)
Ru2A	0.0161 (3)	0.0126 (2)	0.0253 (3)	0.0002 (2)	0.0020 (3)	0.0024 (2)
Ru3A	0.0172 (3)	0.0163 (3)	0.0240 (3)	−0.0009 (2)	0.0032 (3)	0.0005 (2)
As1A	0.0159 (4)	0.0091 (3)	0.0205 (4)	0.0006 (3)	−0.0004 (3)	0.0016 (3)
As2A	0.0155 (4)	0.0090 (3)	0.0222 (4)	0.0004 (3)	−0.0003 (3)	0.0011 (3)
P1A	0.0259 (11)	0.0176 (9)	0.0272 (12)	−0.0021 (8)	0.0064 (9)	−0.0051 (8)
O1A	0.018 (4)	0.041 (4)	0.058 (5)	−0.004 (3)	−0.011 (3)	0.010 (4)
O2A	0.069 (4)	0.0167 (18)	0.055 (3)	−0.002 (2)	−0.006 (3)	0.0006 (19)
O3A	0.037 (4)	0.061 (5)	0.038 (4)	−0.025 (4)	−0.011 (3)	0.013 (4)
O4A	0.022 (3)	0.068 (5)	0.047 (5)	0.021 (3)	−0.006 (3)	−0.010 (4)
O5A	0.032 (4)	0.031 (4)	0.099 (7)	0.010 (3)	0.024 (4)	0.026 (4)
O6A	0.020 (4)	0.054 (4)	0.054 (5)	0.000 (3)	−0.009 (3)	−0.020 (4)
O7A	0.026 (4)	0.046 (4)	0.039 (4)	−0.013 (3)	0.004 (3)	−0.009 (3)
O8A	0.033 (4)	0.023 (3)	0.041 (4)	0.000 (3)	0.011 (3)	0.011 (3)
O9A	0.032 (4)	0.033 (3)	0.036 (4)	−0.006 (3)	−0.006 (3)	0.000 (3)
O10A	0.039 (4)	0.043 (4)	0.038 (4)	0.018 (3)	0.001 (3)	−0.004 (3)
O11A	0.020 (2)	0.0160 (18)	0.052 (3)	−0.0006 (15)	−0.005 (2)	0.0024 (19)
O12A	0.051 (4)	0.024 (3)	0.037 (4)	−0.004 (3)	0.008 (3)	0.002 (3)
C1A	0.014 (4)	0.013 (3)	0.028 (5)	0.002 (3)	0.001 (3)	0.004 (3)
C2A	0.017 (4)	0.017 (4)	0.031 (5)	0.002 (3)	0.001 (4)	−0.001 (3)
C3A	0.020 (4)	0.018 (4)	0.033 (5)	−0.001 (3)	0.001 (4)	0.012 (3)
C4A	0.030 (5)	0.012 (3)	0.041 (6)	−0.007 (3)	−0.004 (4)	0.009 (3)
C5A	0.026 (5)	0.015 (3)	0.032 (5)	0.001 (3)	−0.011 (4)	−0.003 (3)
C6A	0.027 (5)	0.011 (3)	0.029 (5)	0.001 (3)	−0.002 (4)	0.003 (3)
C7A	0.018 (4)	0.015 (3)	0.028 (5)	−0.001 (3)	0.000 (4)	0.005 (3)
C8A	0.023 (4)	0.020 (4)	0.036 (5)	0.003 (3)	−0.009 (4)	−0.001 (3)
C9A	0.034 (5)	0.028 (4)	0.032 (5)	−0.005 (4)	−0.009 (4)	−0.002 (4)
C10A	0.042 (6)	0.036 (5)	0.023 (5)	−0.012 (4)	−0.017 (4)	0.008 (4)
C11A	0.027 (5)	0.046 (6)	0.032 (6)	0.006 (4)	−0.002 (4)	0.015 (5)
C12A	0.026 (5)	0.030 (4)	0.023 (5)	0.005 (4)	−0.005 (4)	0.006 (3)
C13A	0.010 (4)	0.013 (3)	0.026 (4)	0.004 (2)	0.002 (3)	0.001 (3)
C14A	0.019 (4)	0.015 (3)	0.023 (4)	0.004 (3)	0.002 (3)	0.002 (3)
C15A	0.026 (5)	0.011 (3)	0.027 (4)	0.002 (3)	0.003 (3)	0.003 (3)
C16A	0.028 (5)	0.017 (3)	0.035 (5)	−0.002 (3)	−0.002 (4)	−0.002 (3)
C17A	0.032 (5)	0.023 (4)	0.037 (6)	0.003 (3)	−0.006 (4)	−0.006 (4)
C18A	0.026 (5)	0.023 (4)	0.023 (4)	0.000 (3)	0.001 (4)	0.002 (3)
C19A	0.015 (4)	0.017 (3)	0.033 (5)	0.000 (3)	−0.002 (3)	−0.001 (3)
C20A	0.021 (4)	0.015 (3)	0.019 (4)	0.002 (3)	−0.002 (3)	−0.002 (3)
C21A	0.018 (4)	0.014 (3)	0.023 (4)	−0.002 (3)	0.003 (3)	0.001 (3)
C22A	0.035 (5)	0.018 (3)	0.019 (4)	0.008 (3)	0.000 (4)	0.002 (3)
C23A	0.028 (5)	0.021 (4)	0.032 (5)	0.013 (3)	−0.013 (4)	−0.004 (4)
C24A	0.018 (4)	0.025 (4)	0.036 (5)	0.008 (3)	−0.001 (4)	−0.002 (4)
C25A	0.019 (4)	0.017 (3)	0.028 (5)	0.001 (3)	0.003 (3)	−0.002 (3)
C26A	0.031 (5)	0.029 (4)	0.036 (5)	0.014 (4)	0.010 (4)	0.007 (4)
C27A	0.055 (7)	0.033 (5)	0.043 (6)	0.006 (5)	−0.014 (6)	−0.003 (4)
C28A	0.047 (7)	0.035 (5)	0.054 (7)	−0.005 (4)	−0.020 (6)	0.011 (5)
C29A	0.029 (5)	0.036 (5)	0.051 (6)	0.001 (4)	0.009 (5)	0.013 (5)
C30A	0.033 (5)	0.034 (5)	0.029 (5)	0.005 (4)	0.004 (4)	0.005 (4)
C31A	0.030 (5)	0.035 (5)	0.040 (6)	0.008 (4)	−0.003 (5)	−0.004 (4)
C32A	0.028 (5)	0.018 (4)	0.041 (6)	0.001 (3)	0.012 (4)	0.002 (4)
C33A	0.032 (5)	0.048 (6)	0.032 (6)	0.007 (4)	0.000 (4)	−0.010 (5)
C34A	0.033 (6)	0.080 (9)	0.040 (7)	0.021 (6)	0.012 (5)	0.023 (6)
C35A	0.024 (5)	0.060 (7)	0.074 (9)	0.000 (5)	0.016 (6)	0.033 (7)
C36A	0.030 (6)	0.044 (6)	0.101 (11)	−0.019 (5)	0.008 (7)	0.027 (7)
C37A	0.033 (6)	0.038 (5)	0.042 (6)	−0.009 (4)	0.001 (5)	0.006 (5)
C38A	0.021 (4)	0.016 (3)	0.041 (6)	0.002 (3)	0.000 (4)	−0.005 (3)
C39A	0.023 (5)	0.021 (4)	0.035 (5)	−0.005 (3)	0.001 (4)	−0.004 (3)
C40A	0.055 (7)	0.031 (5)	0.030 (5)	0.002 (4)	0.007 (5)	−0.004 (4)
C41A	0.045 (6)	0.038 (5)	0.050 (7)	−0.006 (4)	0.004 (5)	−0.011 (5)
C42A	0.028 (5)	0.033 (5)	0.060 (7)	0.000 (4)	0.007 (5)	−0.015 (4)
C43A	0.046 (6)	0.032 (5)	0.049 (7)	−0.006 (4)	0.010 (5)	−0.008 (4)
C44A	0.028 (5)	0.020 (4)	0.033 (5)	0.005 (3)	0.008 (4)	0.009 (4)
C45A	0.044 (6)	0.021 (4)	0.022 (5)	0.000 (3)	0.006 (4)	0.001 (3)
C46A	0.032 (5)	0.035 (4)	0.021 (5)	−0.017 (4)	−0.004 (4)	0.011 (4)
C47A	0.028 (5)	0.031 (4)	0.029 (5)	0.003 (4)	−0.005 (4)	0.010 (4)
C48A	0.032 (5)	0.025 (4)	0.050 (6)	0.005 (4)	0.022 (5)	0.015 (4)
C49A	0.025 (5)	0.026 (4)	0.038 (6)	0.002 (4)	0.008 (4)	−0.010 (4)
C50A	0.018 (4)	0.026 (4)	0.027 (5)	0.000 (3)	0.009 (4)	−0.004 (3)
C51A	0.025 (5)	0.018 (3)	0.035 (5)	−0.002 (3)	0.004 (4)	−0.005 (3)
C52A	0.016 (4)	0.021 (4)	0.033 (5)	0.001 (3)	−0.001 (4)	0.003 (3)
Ru1B	0.0209 (3)	0.0121 (2)	0.0210 (3)	−0.0001 (2)	−0.0020 (3)	0.0000 (2)
Ru2B	0.0174 (3)	0.0125 (2)	0.0234 (3)	0.0012 (2)	−0.0031 (3)	−0.0005 (2)
Ru3B	0.0188 (3)	0.0156 (3)	0.0199 (3)	0.0014 (2)	−0.0027 (3)	−0.0007 (2)
As1B	0.0158 (4)	0.0100 (3)	0.0212 (4)	0.0000 (3)	−0.0006 (3)	0.0010 (3)
As2B	0.0141 (4)	0.0097 (3)	0.0235 (4)	0.0002 (3)	−0.0014 (3)	0.0014 (3)
P1B	0.0237 (11)	0.0152 (8)	0.0225 (11)	0.0001 (7)	−0.0034 (9)	−0.0016 (8)
O1B	0.026 (3)	0.044 (4)	0.049 (5)	0.014 (3)	0.009 (3)	0.009 (3)
O2B	0.069 (4)	0.0167 (18)	0.055 (3)	−0.002 (2)	−0.006 (3)	0.0006 (19)
O3B	0.023 (4)	0.037 (4)	0.044 (4)	−0.005 (3)	0.004 (3)	0.012 (3)
O4B	0.032 (4)	0.055 (5)	0.033 (4)	−0.004 (3)	0.006 (3)	−0.020 (4)
O5B	0.056 (5)	0.020 (3)	0.052 (5)	0.014 (3)	−0.021 (4)	0.006 (3)
O6B	0.035 (4)	0.052 (4)	0.037 (4)	−0.012 (3)	0.010 (3)	−0.011 (4)
O7B	0.028 (3)	0.031 (3)	0.033 (4)	0.000 (3)	0.007 (3)	−0.002 (3)
O8B	0.049 (4)	0.021 (3)	0.030 (4)	−0.002 (3)	−0.005 (3)	0.008 (3)
O9B	0.026 (4)	0.047 (4)	0.040 (4)	0.016 (3)	−0.002 (3)	−0.005 (3)
O10B	0.030 (3)	0.017 (3)	0.025 (3)	−0.004 (2)	−0.011 (3)	0.004 (2)
O11B	0.020 (2)	0.0160 (18)	0.052 (3)	−0.0006 (15)	−0.005 (2)	0.0024 (19)
O12B	0.027 (3)	0.022 (3)	0.039 (4)	−0.003 (2)	−0.002 (3)	−0.013 (2)
C1B	0.024 (5)	0.006 (3)	0.025 (4)	0.003 (3)	0.000 (3)	0.002 (3)
C2B	0.021 (4)	0.011 (3)	0.037 (5)	−0.003 (3)	−0.004 (4)	0.004 (3)
C3B	0.020 (5)	0.020 (4)	0.051 (7)	0.002 (3)	−0.015 (4)	0.008 (4)
C4B	0.028 (5)	0.022 (4)	0.037 (6)	0.005 (3)	0.003 (4)	0.012 (4)
C5B	0.037 (5)	0.016 (4)	0.038 (6)	0.007 (3)	0.004 (4)	0.002 (4)
C6B	0.024 (4)	0.019 (4)	0.029 (5)	0.004 (3)	0.007 (4)	0.007 (3)
C7B	0.020 (4)	0.020 (4)	0.022 (4)	0.004 (3)	0.000 (3)	0.003 (3)
C8B	0.028 (5)	0.015 (3)	0.033 (5)	−0.001 (3)	−0.002 (4)	−0.003 (3)
C9B	0.051 (6)	0.019 (4)	0.023 (5)	0.004 (4)	−0.001 (4)	0.000 (3)
C10B	0.026 (5)	0.026 (4)	0.036 (6)	0.003 (3)	0.005 (4)	0.012 (4)
C11B	0.019 (4)	0.034 (5)	0.036 (6)	−0.002 (3)	0.000 (4)	0.005 (4)
C12B	0.016 (4)	0.020 (4)	0.030 (5)	−0.001 (3)	−0.001 (4)	0.001 (3)
C13B	0.017 (4)	0.011 (3)	0.031 (5)	−0.003 (3)	−0.005 (3)	0.003 (3)
C14B	0.015 (4)	0.016 (3)	0.029 (5)	−0.003 (3)	0.004 (3)	−0.006 (3)
C15B	0.023 (4)	0.017 (3)	0.030 (5)	0.006 (3)	−0.002 (4)	−0.007 (3)
C16B	0.032 (5)	0.014 (3)	0.039 (5)	0.005 (3)	0.007 (4)	−0.009 (3)
C17B	0.026 (5)	0.021 (4)	0.026 (5)	−0.001 (3)	0.005 (4)	−0.011 (3)
C19B	0.019 (4)	0.014 (3)	0.030 (5)	0.003 (3)	−0.006 (3)	0.000 (3)
C18B	0.025 (5)	0.021 (4)	0.026 (5)	−0.002 (3)	−0.002 (4)	−0.003 (3)
C20B	0.022 (4)	0.011 (3)	0.025 (5)	−0.002 (3)	0.006 (3)	−0.001 (3)
C21B	0.031 (5)	0.014 (3)	0.029 (5)	−0.003 (3)	0.005 (4)	0.000 (3)
C22B	0.047 (6)	0.016 (3)	0.022 (5)	−0.010 (3)	0.005 (4)	0.000 (3)
C23B	0.033 (5)	0.029 (4)	0.034 (6)	−0.005 (4)	0.005 (4)	−0.013 (4)
C24B	0.024 (5)	0.016 (4)	0.043 (6)	−0.001 (3)	0.001 (4)	−0.009 (4)
C25B	0.021 (4)	0.023 (4)	0.031 (5)	−0.001 (3)	0.003 (4)	0.000 (3)
C26B	0.031 (5)	0.020 (4)	0.018 (4)	−0.001 (3)	−0.009 (4)	0.001 (3)
C27B	0.020 (5)	0.039 (5)	0.038 (6)	0.008 (4)	0.005 (4)	0.006 (4)
C28B	0.026 (5)	0.035 (5)	0.061 (8)	0.003 (4)	−0.005 (5)	0.012 (5)
C29B	0.031 (5)	0.029 (4)	0.046 (6)	−0.001 (4)	0.002 (5)	0.012 (4)
C30B	0.028 (5)	0.033 (4)	0.028 (5)	−0.006 (4)	0.001 (4)	−0.001 (4)
C31B	0.030 (5)	0.024 (4)	0.021 (5)	0.000 (3)	0.004 (4)	−0.001 (3)
C32B	0.025 (4)	0.021 (4)	0.026 (5)	0.000 (3)	−0.004 (4)	0.010 (3)
C33B	0.031 (5)	0.026 (4)	0.019 (5)	0.001 (3)	−0.001 (4)	0.000 (3)
C34B	0.040 (5)	0.027 (4)	0.023 (5)	−0.005 (4)	−0.009 (4)	0.003 (3)
C35B	0.022 (4)	0.037 (5)	0.036 (5)	−0.002 (4)	0.005 (4)	0.010 (4)
C36B	0.030 (5)	0.041 (6)	0.051 (7)	0.007 (4)	0.011 (5)	−0.009 (5)
C37B	0.035 (5)	0.029 (4)	0.037 (6)	−0.002 (4)	0.001 (4)	−0.007 (4)
C38B	0.018 (9)	0.016 (5)	0.037 (8)	0.001 (5)	0.007 (7)	−0.009 (5)
C39B	0.022 (8)	0.012 (5)	0.030 (7)	−0.001 (5)	0.015 (6)	0.000 (5)
C40B	0.051 (15)	0.017 (6)	0.038 (9)	0.012 (7)	0.001 (9)	−0.009 (5)
C41B	0.066 (12)	0.010 (5)	0.044 (9)	−0.002 (7)	−0.003 (8)	−0.011 (5)
C42B	0.082 (14)	0.027 (6)	0.069 (11)	0.020 (9)	−0.026 (10)	−0.010 (7)
C43B	0.043 (11)	0.027 (7)	0.054 (9)	0.011 (7)	−0.020 (9)	−0.014 (6)
C44B	0.035 (5)	0.033 (4)	0.009 (4)	0.012 (4)	−0.001 (3)	0.001 (3)
C45B	0.029 (5)	0.018 (3)	0.038 (5)	0.000 (3)	−0.013 (4)	0.006 (4)
C46B	0.030 (5)	0.019 (4)	0.028 (5)	−0.003 (3)	−0.002 (4)	0.008 (3)
C47B	0.028 (5)	0.026 (4)	0.039 (6)	−0.005 (4)	−0.015 (5)	−0.010 (4)
C48B	0.041 (6)	0.020 (4)	0.046 (6)	0.001 (4)	−0.010 (5)	0.000 (4)
C49B	0.025 (5)	0.027 (4)	0.030 (5)	−0.001 (3)	−0.010 (4)	0.000 (4)
C50B	0.027 (4)	0.025 (4)	0.018 (4)	−0.009 (3)	−0.003 (3)	0.004 (3)
C51B	0.025 (5)	0.019 (4)	0.031 (5)	0.004 (3)	−0.008 (4)	−0.009 (3)
C52B	0.033 (5)	0.023 (4)	0.032 (5)	0.002 (4)	−0.013 (4)	0.002 (4)

### Geometric parameters (Å, °)

**Table:**

Ru1A—C45A	1.900 (9)	Ru2B—C48B	1.885 (9)
Ru1A—C44A	1.911 (11)	Ru2B—C47B	1.898 (12)
Ru1A—C46A	1.929 (9)	Ru2B—C49B	1.944 (10)
Ru1A—As1A	2.4301 (12)	Ru2B—As2B	2.4189 (11)
Ru1A—Ru3A	2.8432 (10)	Ru2B—Ru3B	2.8256 (9)
Ru1A—Ru2A	2.8594 (8)	Ru3B—C51B	1.892 (9)
Ru2A—C47A	1.893 (10)	Ru3B—C52B	1.941 (11)
Ru2A—C48A	1.897 (9)	Ru3B—C50B	1.956 (9)
Ru2A—C49A	1.938 (11)	Ru3B—P1B	2.247 (2)
Ru2A—As2A	2.4423 (11)	As1B—C7B	1.943 (9)
Ru2A—Ru3A	2.8513 (10)	As1B—C1B	1.958 (7)
Ru3A—C51A	1.918 (9)	As1B—C13B	1.966 (8)
Ru3A—C50A	1.934 (10)	As2B—C20B	1.940 (8)
Ru3A—C52A	1.955 (9)	As2B—C13B	1.956 (7)
Ru3A—P1A	2.263 (2)	As2B—C14B	1.961 (8)
As1A—C7A	1.926 (9)	P1B—O11B	1.558 (6)
As1A—C1A	1.956 (8)	P1B—O10B	1.608 (6)
As1A—C13A	1.972 (7)	P1B—O12B	1.618 (6)
As2A—C20A	1.945 (8)	O1B—C44B	1.144 (12)
As2A—C14A	1.947 (8)	O2B—C45B	1.156 (10)
As2A—C13A	1.962 (7)	O3B—C46B	1.154 (12)
P1A—O12A	1.583 (7)	O4B—C47B	1.178 (13)
P1A—O11A	1.597 (7)	O5B—C48B	1.145 (11)
P1A—O10A	1.597 (7)	O6B—C49B	1.155 (12)
O1A—C44A	1.150 (12)	O7B—C50B	1.149 (11)
O2A—C45A	1.131 (11)	O8B—C51B	1.158 (11)
O3A—C46A	1.127 (11)	O9B—C52B	1.154 (12)
O4A—C47A	1.152 (11)	O10B—C26B	1.391 (10)
O5A—C48A	1.139 (11)	O11B—C32B	1.440 (10)
O6A—C49A	1.117 (12)	O12B—C38B	1.358 (16)
O7A—C50A	1.129 (11)	O12B—C38C	1.46 (2)
O8A—C51A	1.135 (11)	C1B—C2B	1.358 (13)
O9A—C52A	1.125 (10)	C1B—C6B	1.398 (12)
O10A—C26A	1.413 (10)	C2B—C3B	1.405 (11)
O11A—C32A	1.404 (11)	C2B—H2BA	0.9300
O12A—C38A	1.396 (12)	C3B—C4B	1.378 (14)
C1A—C6A	1.383 (12)	C3B—H3BA	0.9300
C1A—C2A	1.403 (13)	C4B—C5B	1.372 (14)
C2A—C3A	1.391 (11)	C4B—H4BA	0.9300
C2A—H2AA	0.9300	C5B—C6B	1.389 (12)
C3A—C4A	1.395 (13)	C5B—H5BA	0.9300
C3A—H3AA	0.9300	C6B—H6BA	0.9300
C4A—C5A	1.392 (14)	C7B—C12B	1.388 (12)
C4A—H4AA	0.9300	C7B—C8B	1.397 (12)
C5A—C6A	1.405 (12)	C8B—C9B	1.405 (13)
C5A—H5AA	0.9300	C8B—H8BA	0.9300
C6A—H6AA	0.9300	C9B—C10B	1.387 (13)
C7A—C8A	1.397 (12)	C9B—H9BA	0.9300
C7A—C12A	1.398 (12)	C10B—C11B	1.366 (14)
C8A—C9A	1.371 (13)	C10B—H10B	0.9300
C8A—H8AA	0.9300	C11B—C12B	1.383 (14)
C9A—C10A	1.371 (14)	C11B—H11B	0.9300
C9A—H9AA	0.9300	C12B—H12B	0.9300
C10A—C11A	1.406 (15)	C13B—H13C	0.9700
C10A—H10A	0.9300	C13B—H13D	0.9700
C11A—C12A	1.376 (14)	C14B—C19B	1.381 (12)
C11A—H11A	0.9300	C14B—C15B	1.384 (11)
C12A—H12A	0.9300	C15B—C16B	1.385 (11)
C13A—H13A	0.9700	C15B—H15B	0.9300
C13A—H13B	0.9700	C16B—C17B	1.357 (14)
C14A—C15A	1.383 (11)	C16B—H16B	0.9300
C14A—C19A	1.400 (12)	C17B—C18B	1.399 (12)
C15A—C16A	1.380 (12)	C17B—H17B	0.9300
C15A—H15A	0.9300	C19B—C18B	1.398 (12)
C16A—C17A	1.392 (14)	C19B—H19B	0.9300
C16A—H16A	0.9300	C18B—H18B	0.9300
C17A—C18A	1.386 (13)	C20B—C21B	1.385 (11)
C17A—H17A	0.9300	C20B—C25B	1.387 (13)
C18A—C19A	1.381 (12)	C21B—C22B	1.382 (12)
C18A—H18A	0.9300	C21B—H21B	0.9300
C19A—H19A	0.9300	C22B—C23B	1.369 (15)
C20A—C25A	1.374 (12)	C22B—H22B	0.9300
C20A—C21A	1.402 (11)	C23B—C24B	1.366 (14)
C21A—C22A	1.377 (11)	C23B—H23B	0.9300
C21A—H21A	0.9300	C24B—C25B	1.383 (13)
C22A—C23A	1.398 (14)	C24B—H24B	0.9300
C22A—H22A	0.9300	C25B—H25B	0.9300
C23A—C24A	1.399 (13)	C26B—C27B	1.386 (13)
C23A—H23A	0.9300	C26B—C31B	1.395 (13)
C24A—C25A	1.402 (12)	C27B—C28B	1.373 (14)
C24A—H24A	0.9300	C27B—H27B	0.9300
C25A—H25A	0.9300	C28B—C29B	1.399 (16)
C26A—C27A	1.349 (15)	C28B—H28B	0.9300
C26A—C31A	1.401 (14)	C29B—C30B	1.342 (14)
C27A—C28A	1.385 (15)	C29B—H29B	0.9300
C27A—H27A	0.9300	C30B—C31B	1.376 (13)
C28A—C29A	1.375 (15)	C30B—H30B	0.9300
C28A—H28A	0.9300	C31B—H31B	0.9300
C29A—C30A	1.331 (14)	C32B—C37B	1.352 (13)
C29A—H29A	0.9300	C32B—C33B	1.372 (13)
C30A—C31A	1.391 (13)	C33B—C34B	1.385 (14)
C30A—H30A	0.9300	C33B—H33B	0.9300
C31A—H31A	0.9300	C34B—C35B	1.387 (14)
C32A—C37A	1.358 (14)	C34B—H34B	0.9300
C32A—C33A	1.360 (15)	C35B—C36B	1.394 (14)
C33A—C34A	1.401 (16)	C35B—H35B	0.9300
C33A—H33A	0.9300	C36B—C37B	1.399 (14)
C34A—C35A	1.355 (18)	C36B—H36B	0.9300
C34A—H34A	0.9300	C37B—H37B	0.9300
C35A—C36A	1.366 (19)	C38B—C39B	1.375 (16)
C35A—H35A	0.9300	C38B—C43B	1.380 (17)
C36A—C37A	1.415 (15)	C39B—C40B	1.362 (15)
C36A—H36A	0.9300	C39B—H39B	0.9300
C37A—H37A	0.9300	C40B—C41B	1.369 (16)
C38A—C43A	1.369 (13)	C40B—H40B	0.9300
C38A—C39A	1.399 (14)	C41B—C42B	1.423 (19)
C39A—C40A	1.350 (13)	C41B—H41B	0.9300
C39A—H39A	0.9300	C42B—C43B	1.364 (17)
C40A—C41A	1.407 (15)	C42B—H42B	0.9300
C40A—H40A	0.9300	C43B—H43B	0.9300
C41A—C42A	1.374 (14)	C38C—C43C	1.379 (19)
C41A—H41A	0.9300	C38C—C39C	1.380 (19)
C42A—C43A	1.413 (14)	C39C—C40C	1.364 (18)
C42A—H42A	0.9300	C39C—H39C	0.9300
C43A—H43A	0.9300	C40C—C41C	1.370 (19)
Ru1B—C45B	1.892 (8)	C40C—H40C	0.9300
Ru1B—C46B	1.933 (10)	C41C—C42C	1.42 (2)
Ru1B—C44B	1.948 (10)	C41C—H41C	0.9300
Ru1B—As1B	2.4025 (11)	C42C—C43C	1.366 (19)
Ru1B—Ru3B	2.8235 (10)	C42C—H42C	0.9300
Ru1B—Ru2B	2.8613 (8)	C43C—H43C	0.9300
			
C45A—Ru1A—C44A	88.9 (4)	C45B—Ru1B—Ru2B	165.4 (3)
C45A—Ru1A—C46A	95.8 (4)	C46B—Ru1B—Ru2B	89.3 (3)
C44A—Ru1A—C46A	174.4 (4)	C44B—Ru1B—Ru2B	84.6 (3)
C45A—Ru1A—As1A	102.9 (3)	As1B—Ru1B—Ru2B	93.13 (3)
C44A—Ru1A—As1A	96.3 (3)	Ru3B—Ru1B—Ru2B	59.60 (2)
C46A—Ru1A—As1A	85.5 (3)	C48B—Ru2B—C47B	90.3 (5)
C45A—Ru1A—Ru3A	104.9 (3)	C48B—Ru2B—C49B	89.9 (4)
C44A—Ru1A—Ru3A	81.8 (3)	C47B—Ru2B—C49B	175.6 (4)
C46A—Ru1A—Ru3A	94.1 (3)	C48B—Ru2B—As2B	104.6 (3)
As1A—Ru1A—Ru3A	152.08 (3)	C47B—Ru2B—As2B	92.7 (3)
C45A—Ru1A—Ru2A	164.8 (3)	C49B—Ru2B—As2B	91.5 (3)
C44A—Ru1A—Ru2A	86.9 (3)	C48B—Ru2B—Ru3B	102.3 (3)
C46A—Ru1A—Ru2A	87.7 (3)	C47B—Ru2B—Ru3B	96.5 (3)
As1A—Ru1A—Ru2A	92.12 (3)	C49B—Ru2B—Ru3B	79.2 (3)
Ru3A—Ru1A—Ru2A	60.00 (2)	As2B—Ru2B—Ru3B	151.54 (3)
C47A—Ru2A—C48A	91.4 (4)	C48B—Ru2B—Ru1B	160.6 (3)
C47A—Ru2A—C49A	175.2 (4)	C47B—Ru2B—Ru1B	85.7 (3)
C48A—Ru2A—C49A	91.3 (5)	C49B—Ru2B—Ru1B	92.7 (3)
C47A—Ru2A—As2A	91.2 (3)	As2B—Ru2B—Ru1B	94.56 (3)
C48A—Ru2A—As2A	103.7 (3)	Ru3B—Ru2B—Ru1B	59.53 (2)
C49A—Ru2A—As2A	92.0 (3)	C51B—Ru3B—C52B	91.1 (4)
C47A—Ru2A—Ru3A	78.3 (3)	C51B—Ru3B—C50B	91.7 (4)
C48A—Ru2A—Ru3A	101.1 (3)	C52B—Ru3B—C50B	176.9 (4)
C49A—Ru2A—Ru3A	97.4 (3)	C51B—Ru3B—P1B	104.5 (3)
As2A—Ru2A—Ru3A	153.27 (3)	C52B—Ru3B—P1B	93.3 (3)
C47A—Ru2A—Ru1A	88.3 (3)	C50B—Ru3B—P1B	87.2 (2)
C48A—Ru2A—Ru1A	160.4 (3)	C51B—Ru3B—Ru1B	157.0 (3)
C49A—Ru2A—Ru1A	87.7 (3)	C52B—Ru3B—Ru1B	95.7 (3)
As2A—Ru2A—Ru1A	95.90 (3)	C50B—Ru3B—Ru1B	81.2 (2)
Ru3A—Ru2A—Ru1A	59.72 (2)	P1B—Ru3B—Ru1B	96.98 (6)
C51A—Ru3A—C50A	90.9 (4)	C51B—Ru3B—Ru2B	98.6 (3)
C51A—Ru3A—C52A	92.3 (4)	C52B—Ru3B—Ru2B	82.2 (3)
C50A—Ru3A—C52A	176.7 (4)	C50B—Ru3B—Ru2B	96.1 (2)
C51A—Ru3A—P1A	106.4 (3)	P1B—Ru3B—Ru2B	156.56 (7)
C50A—Ru3A—P1A	91.0 (3)	Ru1B—Ru3B—Ru2B	60.86 (2)
C52A—Ru3A—P1A	87.5 (2)	C7B—As1B—C1B	101.5 (3)
C51A—Ru3A—Ru1A	154.3 (3)	C7B—As1B—C13B	106.9 (4)
C50A—Ru3A—Ru1A	96.1 (3)	C1B—As1B—C13B	98.0 (3)
C52A—Ru3A—Ru1A	81.1 (3)	C7B—As1B—Ru1B	117.0 (3)
P1A—Ru3A—Ru1A	98.13 (7)	C1B—As1B—Ru1B	118.5 (3)
C51A—Ru3A—Ru2A	96.8 (3)	C13B—As1B—Ru1B	112.6 (3)
C50A—Ru3A—Ru2A	81.0 (3)	C20B—As2B—C13B	102.7 (3)
C52A—Ru3A—Ru2A	99.1 (3)	C20B—As2B—C14B	97.6 (3)
P1A—Ru3A—Ru2A	155.62 (7)	C13B—As2B—C14B	104.1 (4)
Ru1A—Ru3A—Ru2A	60.28 (2)	C20B—As2B—Ru2B	114.3 (3)
C7A—As1A—C1A	101.5 (4)	C13B—As2B—Ru2B	115.2 (2)
C7A—As1A—C13A	105.6 (4)	C14B—As2B—Ru2B	120.3 (3)
C1A—As1A—C13A	97.4 (3)	O11B—P1B—O10B	97.5 (3)
C7A—As1A—Ru1A	117.0 (3)	O11B—P1B—O12B	102.3 (3)
C1A—As1A—Ru1A	118.7 (3)	O10B—P1B—O12B	103.9 (3)
C13A—As1A—Ru1A	114.0 (3)	O11B—P1B—Ru3B	119.7 (3)
C20A—As2A—C14A	96.3 (3)	O10B—P1B—Ru3B	120.5 (2)
C20A—As2A—C13A	103.7 (3)	O12B—P1B—Ru3B	110.4 (2)
C14A—As2A—C13A	104.8 (4)	C26B—O10B—P1B	123.8 (5)
C20A—As2A—Ru2A	114.5 (3)	C32B—O11B—P1B	130.5 (5)
C14A—As2A—Ru2A	120.7 (3)	C38B—O12B—P1B	130.3 (11)
C13A—As2A—Ru2A	114.2 (2)	C38C—O12B—P1B	119.1 (15)
O12A—P1A—O11A	100.9 (3)	C2B—C1B—C6B	120.6 (8)
O12A—P1A—O10A	97.9 (4)	C2B—C1B—As1B	118.9 (6)
O11A—P1A—O10A	102.0 (4)	C6B—C1B—As1B	120.3 (7)
O12A—P1A—Ru3A	120.3 (3)	C1B—C2B—C3B	119.8 (9)
O11A—P1A—Ru3A	121.7 (2)	C1B—C2B—H2BA	120.1
O10A—P1A—Ru3A	110.3 (3)	C3B—C2B—H2BA	120.1
C26A—O10A—P1A	128.8 (7)	C4B—C3B—C2B	119.7 (9)
C32A—O11A—P1A	125.3 (6)	C4B—C3B—H3BA	120.2
C38A—O12A—P1A	133.4 (6)	C2B—C3B—H3BA	120.2
C6A—C1A—C2A	120.0 (8)	C5B—C4B—C3B	120.3 (9)
C6A—C1A—As1A	121.0 (7)	C5B—C4B—H4BA	119.8
C2A—C1A—As1A	119.0 (6)	C3B—C4B—H4BA	119.8
C3A—C2A—C1A	120.5 (8)	C4B—C5B—C6B	120.4 (9)
C3A—C2A—H2AA	119.8	C4B—C5B—H5BA	119.8
C1A—C2A—H2AA	119.8	C6B—C5B—H5BA	119.8
C2A—C3A—C4A	119.8 (9)	C5B—C6B—C1B	119.1 (9)
C2A—C3A—H3AA	120.1	C5B—C6B—H6BA	120.5
C4A—C3A—H3AA	120.1	C1B—C6B—H6BA	120.5
C5A—C4A—C3A	119.5 (8)	C12B—C7B—C8B	117.7 (8)
C5A—C4A—H4AA	120.2	C12B—C7B—As1B	117.9 (7)
C3A—C4A—H4AA	120.2	C8B—C7B—As1B	124.4 (7)
C4A—C5A—C6A	120.9 (8)	C7B—C8B—C9B	120.1 (8)
C4A—C5A—H5AA	119.6	C7B—C8B—H8BA	120.0
C6A—C5A—H5AA	119.6	C9B—C8B—H8BA	120.0
C1A—C6A—C5A	119.3 (9)	C10B—C9B—C8B	120.4 (9)
C1A—C6A—H6AA	120.3	C10B—C9B—H9BA	119.8
C5A—C6A—H6AA	120.3	C8B—C9B—H9BA	119.8
C8A—C7A—C12A	119.9 (8)	C11B—C10B—C9B	119.6 (10)
C8A—C7A—As1A	123.9 (6)	C11B—C10B—H10B	120.2
C12A—C7A—As1A	116.2 (7)	C9B—C10B—H10B	120.2
C9A—C8A—C7A	119.8 (9)	C10B—C11B—C12B	120.2 (9)
C9A—C8A—H8AA	120.1	C10B—C11B—H11B	119.9
C7A—C8A—H8AA	120.1	C12B—C11B—H11B	119.9
C8A—C9A—C10A	120.8 (9)	C11B—C12B—C7B	122.1 (9)
C8A—C9A—H9AA	119.6	C11B—C12B—H12B	118.9
C10A—C9A—H9AA	119.6	C7B—C12B—H12B	118.9
C9A—C10A—C11A	120.0 (9)	As2B—C13B—As1B	112.0 (4)
C9A—C10A—H10A	120.0	As2B—C13B—H13C	109.2
C11A—C10A—H10A	120.0	As1B—C13B—H13C	109.2
C12A—C11A—C10A	119.8 (9)	As2B—C13B—H13D	109.2
C12A—C11A—H11A	120.1	As1B—C13B—H13D	109.2
C10A—C11A—H11A	120.1	H13C—C13B—H13D	107.9
C11A—C12A—C7A	119.7 (9)	C19B—C14B—C15B	119.4 (8)
C11A—C12A—H12A	120.2	C19B—C14B—As2B	122.0 (6)
C7A—C12A—H12A	120.2	C15B—C14B—As2B	118.2 (7)
As2A—C13A—As1A	111.3 (4)	C14B—C15B—C16B	121.2 (9)
As2A—C13A—H13A	109.4	C14B—C15B—H15B	119.4
As1A—C13A—H13A	109.4	C16B—C15B—H15B	119.4
As2A—C13A—H13B	109.4	C17B—C16B—C15B	120.0 (8)
As1A—C13A—H13B	109.4	C17B—C16B—H16B	120.0
H13A—C13A—H13B	108.0	C15B—C16B—H16B	120.0
C15A—C14A—C19A	119.1 (8)	C16B—C17B—C18B	119.8 (8)
C15A—C14A—As2A	118.3 (6)	C16B—C17B—H17B	120.1
C19A—C14A—As2A	122.0 (6)	C18B—C17B—H17B	120.1
C16A—C15A—C14A	120.5 (8)	C14B—C19B—C18B	119.3 (8)
C16A—C15A—H15A	119.8	C14B—C19B—H19B	120.3
C14A—C15A—H15A	119.8	C18B—C19B—H19B	120.3
C15A—C16A—C17A	120.0 (8)	C19B—C18B—C17B	120.3 (8)
C15A—C16A—H16A	120.0	C19B—C18B—H18B	119.9
C17A—C16A—H16A	120.0	C17B—C18B—H18B	119.9
C18A—C17A—C16A	120.2 (9)	C21B—C20B—C25B	117.8 (8)
C18A—C17A—H17A	119.9	C21B—C20B—As2B	118.0 (7)
C16A—C17A—H17A	119.9	C25B—C20B—As2B	124.0 (6)
C19A—C18A—C17A	119.4 (9)	C22B—C21B—C20B	122.2 (9)
C19A—C18A—H18A	120.3	C22B—C21B—H21B	118.9
C17A—C18A—H18A	120.3	C20B—C21B—H21B	118.9
C18A—C19A—C14A	120.8 (8)	C23B—C22B—C21B	118.6 (9)
C18A—C19A—H19A	119.6	C23B—C22B—H22B	120.7
C14A—C19A—H19A	119.6	C21B—C22B—H22B	120.7
C25A—C20A—C21A	121.1 (8)	C24B—C23B—C22B	120.6 (9)
C25A—C20A—As2A	123.1 (6)	C24B—C23B—H23B	119.7
C21A—C20A—As2A	115.5 (6)	C22B—C23B—H23B	119.7
C22A—C21A—C20A	119.2 (8)	C23B—C24B—C25B	120.7 (9)
C22A—C21A—H21A	120.4	C23B—C24B—H24B	119.7
C20A—C21A—H21A	120.4	C25B—C24B—H24B	119.7
C21A—C22A—C23A	121.0 (8)	C24B—C25B—C20B	120.1 (8)
C21A—C22A—H22A	119.5	C24B—C25B—H25B	119.9
C23A—C22A—H22A	119.5	C20B—C25B—H25B	119.9
C22A—C23A—C24A	119.0 (8)	C27B—C26B—O10B	117.0 (9)
C22A—C23A—H23A	120.5	C27B—C26B—C31B	120.7 (9)
C24A—C23A—H23A	120.5	O10B—C26B—C31B	122.3 (8)
C23A—C24A—C25A	120.2 (9)	C28B—C27B—C26B	118.9 (10)
C23A—C24A—H24A	119.9	C28B—C27B—H27B	120.6
C25A—C24A—H24A	119.9	C26B—C27B—H27B	120.6
C20A—C25A—C24A	119.4 (8)	C27B—C28B—C29B	119.8 (10)
C20A—C25A—H25A	120.3	C27B—C28B—H28B	120.1
C24A—C25A—H25A	120.3	C29B—C28B—H28B	120.1
C27A—C26A—C31A	121.5 (9)	C30B—C29B—C28B	121.0 (10)
C27A—C26A—O10A	114.8 (9)	C30B—C29B—H29B	119.5
C31A—C26A—O10A	123.7 (9)	C28B—C29B—H29B	119.5
C26A—C27A—C28A	119.2 (10)	C29B—C30B—C31B	120.5 (10)
C26A—C27A—H27A	120.4	C29B—C30B—H30B	119.7
C28A—C27A—H27A	120.4	C31B—C30B—H30B	119.7
C29A—C28A—C27A	120.1 (10)	C30B—C31B—C26B	119.1 (9)
C29A—C28A—H28A	120.0	C30B—C31B—H31B	120.4
C27A—C28A—H28A	120.0	C26B—C31B—H31B	120.4
C30A—C29A—C28A	120.4 (10)	C37B—C32B—C33B	121.2 (9)
C30A—C29A—H29A	119.8	C37B—C32B—O11B	120.4 (8)
C28A—C29A—H29A	119.8	C33B—C32B—O11B	118.3 (8)
C29A—C30A—C31A	121.7 (10)	C32B—C33B—C34B	119.0 (9)
C29A—C30A—H30A	119.1	C32B—C33B—H33B	120.5
C31A—C30A—H30A	119.1	C34B—C33B—H33B	120.5
C30A—C31A—C26A	117.1 (10)	C33B—C34B—C35B	120.8 (9)
C30A—C31A—H31A	121.4	C33B—C34B—H34B	119.6
C26A—C31A—H31A	121.4	C35B—C34B—H34B	119.6
C37A—C32A—C33A	122.2 (10)	C34B—C35B—C36B	119.6 (9)
C37A—C32A—O11A	120.1 (10)	C34B—C35B—H35B	120.2
C33A—C32A—O11A	117.7 (9)	C36B—C35B—H35B	120.2
C32A—C33A—C34A	118.8 (11)	C35B—C36B—C37B	118.4 (9)
C32A—C33A—H33A	120.6	C35B—C36B—H36B	120.8
C34A—C33A—H33A	120.6	C37B—C36B—H36B	120.8
C35A—C34A—C33A	120.6 (12)	C32B—C37B—C36B	121.0 (9)
C35A—C34A—H34A	119.7	C32B—C37B—H37B	119.5
C33A—C34A—H34A	119.7	C36B—C37B—H37B	119.5
C34A—C35A—C36A	119.8 (11)	O12B—C38B—C39B	121.9 (13)
C34A—C35A—H35A	120.1	O12B—C38B—C43B	117.7 (14)
C36A—C35A—H35A	120.1	C39B—C38B—C43B	120.5 (12)
C35A—C36A—C37A	120.6 (11)	C40B—C39B—C38B	119.2 (12)
C35A—C36A—H36A	119.7	C40B—C39B—H39B	120.4
C37A—C36A—H36A	119.7	C38B—C39B—H39B	120.4
C32A—C37A—C36A	117.9 (11)	C39B—C40B—C41B	121.7 (14)
C32A—C37A—H37A	121.0	C39B—C40B—H40B	119.1
C36A—C37A—H37A	121.0	C41B—C40B—H40B	119.1
C43A—C38A—O12A	115.3 (9)	C40B—C41B—C42B	118.1 (13)
C43A—C38A—C39A	120.7 (9)	C40B—C41B—H41B	120.9
O12A—C38A—C39A	123.7 (8)	C42B—C41B—H41B	120.9
C40A—C39A—C38A	120.8 (9)	C43B—C42B—C41B	118.5 (14)
C40A—C39A—H39A	119.6	C43B—C42B—H42B	120.7
C38A—C39A—H39A	119.6	C41B—C42B—H42B	120.7
C39A—C40A—C41A	118.8 (10)	C42B—C43B—C38B	120.0 (14)
C39A—C40A—H40A	120.6	C42B—C43B—H43B	120.0
C41A—C40A—H40A	120.6	C38B—C43B—H43B	120.0
C42A—C41A—C40A	121.6 (10)	C43C—C38C—C39C	119.4 (18)
C42A—C41A—H41A	119.2	C43C—C38C—O12B	122.1 (18)
C40A—C41A—H41A	119.2	C39C—C38C—O12B	118.5 (17)
C41A—C42A—C43A	118.6 (9)	C40C—C39C—C38C	118.5 (18)
C41A—C42A—H42A	120.7	C40C—C39C—H39C	120.8
C43A—C42A—H42A	120.7	C38C—C39C—H39C	120.8
C38A—C43A—C42A	119.3 (10)	C39C—C40C—C41C	121 (2)
C38A—C43A—H43A	120.3	C39C—C40C—H40C	119.5
C42A—C43A—H43A	120.3	C41C—C40C—H40C	119.5
O1A—C44A—Ru1A	173.7 (8)	C40C—C41C—C42C	117.0 (18)
O2A—C45A—Ru1A	176.5 (10)	C40C—C41C—H41C	121.5
O3A—C46A—Ru1A	172.6 (8)	C42C—C41C—H41C	121.5
O4A—C47A—Ru2A	172.7 (9)	C43C—C42C—C41C	120.1 (17)
O5A—C48A—Ru2A	177.1 (10)	C43C—C42C—H42C	120.0
O6A—C49A—Ru2A	171.9 (9)	C41C—C42C—H42C	120.0
O7A—C50A—Ru3A	172.7 (8)	C42C—C43C—C38C	120.0 (18)
O8A—C51A—Ru3A	176.1 (8)	C42C—C43C—H43C	120.0
O9A—C52A—Ru3A	173.0 (8)	C38C—C43C—H43C	120.0
C45B—Ru1B—C46B	88.0 (4)	O1B—C44B—Ru1B	174.0 (8)
C45B—Ru1B—C44B	97.7 (4)	O2B—C45B—Ru1B	176.7 (10)
C46B—Ru1B—C44B	173.8 (4)	O3B—C46B—Ru1B	174.8 (8)
C45B—Ru1B—As1B	101.4 (3)	O4B—C47B—Ru2B	173.4 (8)
C46B—Ru1B—As1B	95.1 (3)	O5B—C48B—Ru2B	177.0 (10)
C44B—Ru1B—As1B	86.2 (3)	O6B—C49B—Ru2B	173.3 (8)
C45B—Ru1B—Ru3B	105.8 (3)	O7B—C50B—Ru3B	173.7 (7)
C46B—Ru1B—Ru3B	82.5 (3)	O8B—C51B—Ru3B	176.1 (8)
C44B—Ru1B—Ru3B	93.6 (2)	O9B—C52B—Ru3B	173.0 (9)
As1B—Ru1B—Ru3B	152.57 (3)		
			
C45A—Ru1A—Ru2A—C47A	85.1 (12)	Ru3B—Ru1B—Ru2B—C47B	100.1 (3)
C44A—Ru1A—Ru2A—C47A	159.7 (4)	C45B—Ru1B—Ru2B—C49B	−78.3 (13)
C46A—Ru1A—Ru2A—C47A	−18.7 (4)	C46B—Ru1B—Ru2B—C49B	−157.5 (4)
As1A—Ru1A—Ru2A—C47A	−104.1 (3)	C44B—Ru1B—Ru2B—C49B	21.6 (4)
Ru3A—Ru1A—Ru2A—C47A	77.4 (3)	As1B—Ru1B—Ru2B—C49B	107.5 (3)
C45A—Ru1A—Ru2A—C48A	−4.5 (16)	Ru3B—Ru1B—Ru2B—C49B	−75.8 (3)
C44A—Ru1A—Ru2A—C48A	70.0 (11)	C45B—Ru1B—Ru2B—As2B	−170.1 (13)
C46A—Ru1A—Ru2A—C48A	−108.3 (11)	C46B—Ru1B—Ru2B—As2B	110.8 (3)
As1A—Ru1A—Ru2A—C48A	166.2 (11)	C44B—Ru1B—Ru2B—As2B	−70.1 (3)
Ru3A—Ru1A—Ru2A—C48A	−12.2 (11)	As1B—Ru1B—Ru2B—As2B	15.70 (4)
C45A—Ru1A—Ru2A—C49A	−92.2 (12)	Ru3B—Ru1B—Ru2B—As2B	−167.52 (4)
C44A—Ru1A—Ru2A—C49A	−17.6 (4)	C45B—Ru1B—Ru2B—Ru3B	−2.5 (13)
C46A—Ru1A—Ru2A—C49A	164.0 (4)	C46B—Ru1B—Ru2B—Ru3B	−81.7 (3)
As1A—Ru1A—Ru2A—C49A	78.6 (3)	C44B—Ru1B—Ru2B—Ru3B	97.4 (3)
Ru3A—Ru1A—Ru2A—C49A	−99.9 (3)	As1B—Ru1B—Ru2B—Ru3B	−176.78 (4)
C45A—Ru1A—Ru2A—As2A	176.1 (12)	C45B—Ru1B—Ru3B—C51B	−151.8 (8)
C44A—Ru1A—Ru2A—As2A	−109.3 (3)	C46B—Ru1B—Ru3B—C51B	122.5 (8)
C46A—Ru1A—Ru2A—As2A	72.3 (3)	C44B—Ru1B—Ru3B—C51B	−52.7 (7)
As1A—Ru1A—Ru2A—As2A	−13.14 (4)	As1B—Ru1B—Ru3B—C51B	35.9 (7)
Ru3A—Ru1A—Ru2A—As2A	168.37 (4)	Ru2B—Ru1B—Ru3B—C51B	28.9 (7)
C45A—Ru1A—Ru2A—Ru3A	7.7 (12)	C45B—Ru1B—Ru3B—C52B	101.6 (4)
C44A—Ru1A—Ru2A—Ru3A	82.3 (3)	C46B—Ru1B—Ru3B—C52B	15.8 (4)
C46A—Ru1A—Ru2A—Ru3A	−96.1 (3)	C44B—Ru1B—Ru3B—C52B	−159.3 (4)
As1A—Ru1A—Ru2A—Ru3A	178.49 (4)	As1B—Ru1B—Ru3B—C52B	−70.8 (3)
C45A—Ru1A—Ru3A—C51A	153.0 (7)	Ru2B—Ru1B—Ru3B—C52B	−77.8 (3)
C44A—Ru1A—Ru3A—C51A	−120.3 (7)	C45B—Ru1B—Ru3B—C50B	−78.5 (4)
C46A—Ru1A—Ru3A—C51A	55.8 (7)	C46B—Ru1B—Ru3B—C50B	−164.3 (4)
As1A—Ru1A—Ru3A—C51A	−32.4 (6)	C44B—Ru1B—Ru3B—C50B	20.5 (3)
Ru2A—Ru1A—Ru3A—C51A	−29.1 (6)	As1B—Ru1B—Ru3B—C50B	109.1 (2)
C45A—Ru1A—Ru3A—C50A	−101.9 (4)	Ru2B—Ru1B—Ru3B—C50B	102.1 (2)
C44A—Ru1A—Ru3A—C50A	−15.2 (4)	C45B—Ru1B—Ru3B—P1B	7.5 (3)
C46A—Ru1A—Ru3A—C50A	161.0 (4)	C46B—Ru1B—Ru3B—P1B	−78.3 (3)
As1A—Ru1A—Ru3A—C50A	72.8 (3)	C44B—Ru1B—Ru3B—P1B	106.6 (3)
Ru2A—Ru1A—Ru3A—C50A	76.0 (3)	As1B—Ru1B—Ru3B—P1B	−164.88 (10)
C45A—Ru1A—Ru3A—C52A	76.2 (4)	Ru2B—Ru1B—Ru3B—P1B	−171.88 (7)
C44A—Ru1A—Ru3A—C52A	162.9 (4)	C45B—Ru1B—Ru3B—Ru2B	179.3 (3)
C46A—Ru1A—Ru3A—C52A	−20.9 (4)	C46B—Ru1B—Ru3B—Ru2B	93.6 (3)
As1A—Ru1A—Ru3A—C52A	−109.1 (2)	C44B—Ru1B—Ru3B—Ru2B	−81.6 (3)
Ru2A—Ru1A—Ru3A—C52A	−105.9 (2)	As1B—Ru1B—Ru3B—Ru2B	7.00 (8)
C45A—Ru1A—Ru3A—P1A	−10.0 (3)	C48B—Ru2B—Ru3B—C51B	18.2 (5)
C44A—Ru1A—Ru3A—P1A	76.7 (3)	C47B—Ru2B—Ru3B—C51B	109.9 (4)
C46A—Ru1A—Ru3A—P1A	−107.1 (3)	C49B—Ru2B—Ru3B—C51B	−69.3 (4)
As1A—Ru1A—Ru3A—P1A	164.70 (10)	As2B—Ru2B—Ru3B—C51B	−142.1 (3)
Ru2A—Ru1A—Ru3A—P1A	167.93 (7)	Ru1B—Ru2B—Ru3B—C51B	−169.0 (3)
C45A—Ru1A—Ru3A—Ru2A	−177.9 (3)	C48B—Ru2B—Ru3B—C52B	−71.8 (5)
C44A—Ru1A—Ru3A—Ru2A	−91.2 (3)	C47B—Ru2B—Ru3B—C52B	19.9 (4)
C46A—Ru1A—Ru3A—Ru2A	85.0 (3)	C49B—Ru2B—Ru3B—C52B	−159.2 (4)
As1A—Ru1A—Ru3A—Ru2A	−3.23 (8)	As2B—Ru2B—Ru3B—C52B	127.9 (3)
C47A—Ru2A—Ru3A—C51A	72.7 (4)	Ru1B—Ru2B—Ru3B—C52B	101.0 (3)
C48A—Ru2A—Ru3A—C51A	−16.4 (5)	C48B—Ru2B—Ru3B—C50B	110.9 (4)
C49A—Ru2A—Ru3A—C51A	−109.2 (4)	C47B—Ru2B—Ru3B—C50B	−157.4 (4)
As2A—Ru2A—Ru3A—C51A	141.3 (3)	C49B—Ru2B—Ru3B—C50B	23.4 (4)
Ru1A—Ru2A—Ru3A—C51A	167.7 (3)	As2B—Ru2B—Ru3B—C50B	−49.5 (2)
C47A—Ru2A—Ru3A—C50A	162.6 (4)	Ru1B—Ru2B—Ru3B—C50B	−76.3 (2)
C48A—Ru2A—Ru3A—C50A	73.5 (5)	C48B—Ru2B—Ru3B—P1B	−152.1 (4)
C49A—Ru2A—Ru3A—C50A	−19.4 (4)	C47B—Ru2B—Ru3B—P1B	−60.5 (3)
As2A—Ru2A—Ru3A—C50A	−128.9 (3)	C49B—Ru2B—Ru3B—P1B	120.4 (3)
Ru1A—Ru2A—Ru3A—C50A	−102.4 (3)	As2B—Ru2B—Ru3B—P1B	47.5 (2)
C47A—Ru2A—Ru3A—C52A	−20.7 (4)	Ru1B—Ru2B—Ru3B—P1B	20.64 (17)
C48A—Ru2A—Ru3A—C52A	−109.9 (4)	C48B—Ru2B—Ru3B—Ru1B	−172.8 (4)
C49A—Ru2A—Ru3A—C52A	157.3 (4)	C47B—Ru2B—Ru3B—Ru1B	−81.1 (3)
As2A—Ru2A—Ru3A—C52A	47.8 (3)	C49B—Ru2B—Ru3B—Ru1B	99.7 (3)
Ru1A—Ru2A—Ru3A—C52A	74.2 (2)	As2B—Ru2B—Ru3B—Ru1B	26.88 (8)
C47A—Ru2A—Ru3A—P1A	−125.1 (3)	C45B—Ru1B—As1B—C7B	−86.5 (4)
C48A—Ru2A—Ru3A—P1A	145.7 (4)	C46B—Ru1B—As1B—C7B	2.4 (4)
C49A—Ru2A—Ru3A—P1A	52.9 (3)	C44B—Ru1B—As1B—C7B	176.3 (4)
As2A—Ru2A—Ru3A—P1A	−56.58 (19)	Ru3B—Ru1B—As1B—C7B	86.0 (3)
Ru1A—Ru2A—Ru3A—P1A	−30.11 (16)	Ru2B—Ru1B—As1B—C7B	92.0 (3)
C47A—Ru2A—Ru3A—Ru1A	−95.0 (3)	C45B—Ru1B—As1B—C1B	35.6 (4)
C48A—Ru2A—Ru3A—Ru1A	175.9 (4)	C46B—Ru1B—As1B—C1B	124.6 (4)
C49A—Ru2A—Ru3A—Ru1A	83.0 (3)	C44B—Ru1B—As1B—C1B	−61.5 (4)
As2A—Ru2A—Ru3A—Ru1A	−26.47 (8)	Ru3B—Ru1B—As1B—C1B	−151.9 (3)
C45A—Ru1A—As1A—C7A	84.8 (4)	Ru2B—Ru1B—As1B—C1B	−145.9 (3)
C44A—Ru1A—As1A—C7A	−5.5 (4)	C45B—Ru1B—As1B—C13B	149.0 (4)
C46A—Ru1A—As1A—C7A	179.8 (4)	C46B—Ru1B—As1B—C13B	−122.0 (4)
Ru3A—Ru1A—As1A—C7A	−89.9 (3)	C44B—Ru1B—As1B—C13B	51.9 (4)
Ru2A—Ru1A—As1A—C7A	−92.7 (3)	Ru3B—Ru1B—As1B—C13B	−38.5 (3)
C45A—Ru1A—As1A—C1A	−37.4 (4)	Ru2B—Ru1B—As1B—C13B	−32.5 (3)
C44A—Ru1A—As1A—C1A	−127.8 (4)	C48B—Ru2B—As2B—C20B	−62.6 (4)
C46A—Ru1A—As1A—C1A	57.5 (4)	C47B—Ru2B—As2B—C20B	−153.6 (4)
Ru3A—Ru1A—As1A—C1A	147.9 (3)	C49B—Ru2B—As2B—C20B	27.7 (4)
Ru2A—Ru1A—As1A—C1A	145.1 (3)	Ru3B—Ru2B—As2B—C20B	97.5 (3)
C45A—Ru1A—As1A—C13A	−151.3 (4)	Ru1B—Ru2B—As2B—C20B	120.5 (2)
C44A—Ru1A—As1A—C13A	118.3 (4)	C48B—Ru2B—As2B—C13B	178.8 (5)
C46A—Ru1A—As1A—C13A	−56.4 (4)	C47B—Ru2B—As2B—C13B	87.8 (4)
Ru3A—Ru1A—As1A—C13A	34.0 (3)	C49B—Ru2B—As2B—C13B	−90.9 (4)
Ru2A—Ru1A—As1A—C13A	31.2 (3)	Ru3B—Ru2B—As2B—C13B	−21.0 (3)
C47A—Ru2A—As2A—C20A	−36.1 (4)	Ru1B—Ru2B—As2B—C13B	2.0 (3)
C48A—Ru2A—As2A—C20A	55.7 (5)	C48B—Ru2B—As2B—C14B	53.0 (5)
C49A—Ru2A—As2A—C20A	147.5 (4)	C47B—Ru2B—As2B—C14B	−38.0 (4)
Ru3A—Ru2A—As2A—C20A	−101.8 (3)	C49B—Ru2B—As2B—C14B	143.2 (4)
Ru1A—Ru2A—As2A—C20A	−124.5 (3)	Ru3B—Ru2B—As2B—C14B	−146.9 (3)
C47A—Ru2A—As2A—C14A	−150.3 (4)	Ru1B—Ru2B—As2B—C14B	−123.9 (3)
C48A—Ru2A—As2A—C14A	−58.5 (5)	C51B—Ru3B—P1B—O11B	−146.9 (4)
C49A—Ru2A—As2A—C14A	33.3 (4)	C52B—Ru3B—P1B—O11B	−54.9 (4)
Ru3A—Ru2A—As2A—C14A	144.0 (3)	C50B—Ru3B—P1B—O11B	122.1 (4)
Ru1A—Ru2A—As2A—C14A	121.3 (3)	Ru1B—Ru3B—P1B—O11B	41.3 (3)
C47A—Ru2A—As2A—C13A	83.3 (4)	Ru2B—Ru3B—P1B—O11B	23.2 (4)
C48A—Ru2A—As2A—C13A	175.1 (5)	C51B—Ru3B—P1B—O10B	−26.3 (4)
C49A—Ru2A—As2A—C13A	−93.1 (4)	C52B—Ru3B—P1B—O10B	65.7 (4)
Ru3A—Ru2A—As2A—C13A	17.6 (3)	C50B—Ru3B—P1B—O10B	−117.3 (4)
Ru1A—Ru2A—As2A—C13A	−5.2 (3)	Ru1B—Ru3B—P1B—O10B	161.9 (3)
C51A—Ru3A—P1A—O12A	−124.1 (4)	Ru2B—Ru3B—P1B—O10B	143.8 (3)
C50A—Ru3A—P1A—O12A	144.6 (4)	C51B—Ru3B—P1B—O12B	94.8 (4)
C52A—Ru3A—P1A—O12A	−32.4 (4)	C52B—Ru3B—P1B—O12B	−173.1 (4)
Ru1A—Ru3A—P1A—O12A	48.2 (3)	C50B—Ru3B—P1B—O12B	3.8 (4)
Ru2A—Ru3A—P1A—O12A	74.3 (4)	Ru1B—Ru3B—P1B—O12B	−76.9 (3)
C51A—Ru3A—P1A—O11A	4.0 (4)	Ru2B—Ru3B—P1B—O12B	−95.0 (3)
C50A—Ru3A—P1A—O11A	−87.3 (4)	O11B—P1B—O10B—C26B	163.7 (7)
C52A—Ru3A—P1A—O11A	95.7 (4)	O12B—P1B—O10B—C26B	−91.6 (7)
Ru1A—Ru3A—P1A—O11A	176.3 (3)	Ru3B—P1B—O10B—C26B	32.6 (8)
Ru2A—Ru3A—P1A—O11A	−157.5 (3)	O10B—P1B—O11B—C32B	−8.4 (8)
C51A—Ru3A—P1A—O10A	123.2 (4)	O12B—P1B—O11B—C32B	−114.5 (7)
C50A—Ru3A—P1A—O10A	31.9 (4)	Ru3B—P1B—O11B—C32B	123.2 (7)
C52A—Ru3A—P1A—O10A	−145.1 (4)	O11B—P1B—O12B—C38B	43.5 (12)
Ru1A—Ru3A—P1A—O10A	−64.4 (3)	O10B—P1B—O12B—C38B	−57.5 (12)
Ru2A—Ru3A—P1A—O10A	−38.3 (4)	Ru3B—P1B—O12B—C38B	172.0 (11)
O12A—P1A—O10A—C26A	63.0 (9)	O11B—P1B—O12B—C38C	39.3 (12)
O11A—P1A—O10A—C26A	−39.9 (9)	O10B—P1B—O12B—C38C	−61.8 (12)
Ru3A—P1A—O10A—C26A	−170.5 (7)	Ru3B—P1B—O12B—C38C	167.7 (11)
O12A—P1A—O11A—C32A	147.5 (8)	C7B—As1B—C1B—C2B	−50.7 (8)
O10A—P1A—O11A—C32A	−111.9 (8)	C13B—As1B—C1B—C2B	58.4 (8)
Ru3A—P1A—O11A—C32A	11.3 (9)	Ru1B—As1B—C1B—C2B	179.7 (6)
O11A—P1A—O12A—C38A	−36.0 (9)	C7B—As1B—C1B—C6B	134.4 (7)
O10A—P1A—O12A—C38A	−140.0 (8)	C13B—As1B—C1B—C6B	−116.5 (7)
Ru3A—P1A—O12A—C38A	101.0 (8)	Ru1B—As1B—C1B—C6B	4.7 (8)
C7A—As1A—C1A—C6A	−131.5 (7)	C6B—C1B—C2B—C3B	−1.3 (13)
C13A—As1A—C1A—C6A	120.8 (7)	As1B—C1B—C2B—C3B	−176.2 (7)
Ru1A—As1A—C1A—C6A	−1.8 (8)	C1B—C2B—C3B—C4B	0.6 (14)
C7A—As1A—C1A—C2A	49.3 (7)	C2B—C3B—C4B—C5B	−0.9 (15)
C13A—As1A—C1A—C2A	−58.3 (7)	C3B—C4B—C5B—C6B	1.8 (15)
Ru1A—As1A—C1A—C2A	179.1 (6)	C4B—C5B—C6B—C1B	−2.4 (14)
C6A—C1A—C2A—C3A	−1.4 (13)	C2B—C1B—C6B—C5B	2.2 (13)
As1A—C1A—C2A—C3A	177.8 (6)	As1B—C1B—C6B—C5B	177.0 (7)
C1A—C2A—C3A—C4A	1.5 (13)	C1B—As1B—C7B—C12B	−79.3 (7)
C2A—C3A—C4A—C5A	−1.7 (13)	C13B—As1B—C7B—C12B	178.6 (6)
C3A—C4A—C5A—C6A	1.8 (13)	Ru1B—As1B—C7B—C12B	51.3 (7)
C2A—C1A—C6A—C5A	1.5 (13)	C1B—As1B—C7B—C8B	102.7 (8)
As1A—C1A—C6A—C5A	−177.7 (6)	C13B—As1B—C7B—C8B	0.6 (8)
C4A—C5A—C6A—C1A	−1.7 (13)	Ru1B—As1B—C7B—C8B	−126.8 (7)
C1A—As1A—C7A—C8A	−99.9 (8)	C12B—C7B—C8B—C9B	0.5 (13)
C13A—As1A—C7A—C8A	1.2 (8)	As1B—C7B—C8B—C9B	178.5 (7)
Ru1A—As1A—C7A—C8A	129.3 (7)	C7B—C8B—C9B—C10B	−0.8 (14)
C1A—As1A—C7A—C12A	81.3 (7)	C8B—C9B—C10B—C11B	1.2 (14)
C13A—As1A—C7A—C12A	−177.5 (7)	C9B—C10B—C11B—C12B	−1.3 (14)
Ru1A—As1A—C7A—C12A	−49.5 (7)	C10B—C11B—C12B—C7B	1.0 (14)
C12A—C7A—C8A—C9A	1.4 (14)	C8B—C7B—C12B—C11B	−0.6 (13)
As1A—C7A—C8A—C9A	−177.3 (7)	As1B—C7B—C12B—C11B	−178.8 (7)
C7A—C8A—C9A—C10A	−1.1 (15)	C20B—As2B—C13B—As1B	−149.4 (4)
C8A—C9A—C10A—C11A	0.3 (15)	C14B—As2B—C13B—As1B	109.3 (5)
C9A—C10A—C11A—C12A	0.1 (15)	Ru2B—As2B—C13B—As1B	−24.6 (5)
C10A—C11A—C12A—C7A	0.2 (15)	C7B—As1B—C13B—As2B	−90.3 (5)
C8A—C7A—C12A—C11A	−0.9 (14)	C1B—As1B—C13B—As2B	165.0 (5)
As1A—C7A—C12A—C11A	177.8 (7)	Ru1B—As1B—C13B—As2B	39.5 (5)
C20A—As2A—C13A—As1A	152.3 (4)	C20B—As2B—C14B—C19B	−75.1 (7)
C14A—As2A—C13A—As1A	−107.3 (4)	C13B—As2B—C14B—C19B	30.1 (8)
Ru2A—As2A—C13A—As1A	27.0 (5)	Ru2B—As2B—C14B—C19B	160.9 (6)
C7A—As1A—C13A—As2A	89.5 (5)	C20B—As2B—C14B—C15B	97.3 (7)
C1A—As1A—C13A—As2A	−166.3 (4)	C13B—As2B—C14B—C15B	−157.5 (7)
Ru1A—As1A—C13A—As2A	−40.3 (5)	Ru2B—As2B—C14B—C15B	−26.6 (8)
C20A—As2A—C14A—C15A	−94.7 (7)	C19B—C14B—C15B—C16B	1.6 (14)
C13A—As2A—C14A—C15A	159.2 (6)	As2B—C14B—C15B—C16B	−171.1 (7)
Ru2A—As2A—C14A—C15A	28.6 (7)	C14B—C15B—C16B—C17B	−3.0 (15)
C20A—As2A—C14A—C19A	76.5 (7)	C15B—C16B—C17B—C18B	2.1 (14)
C13A—As2A—C14A—C19A	−29.6 (7)	C15B—C14B—C19B—C18B	0.7 (13)
Ru2A—As2A—C14A—C19A	−160.2 (6)	As2B—C14B—C19B—C18B	173.1 (6)
C19A—C14A—C15A—C16A	1.2 (13)	C14B—C19B—C18B—C17B	−1.6 (13)
As2A—C14A—C15A—C16A	172.7 (7)	C16B—C17B—C18B—C19B	0.1 (14)
C14A—C15A—C16A—C17A	−1.0 (14)	C13B—As2B—C20B—C21B	164.8 (7)
C15A—C16A—C17A—C18A	0.4 (14)	C14B—As2B—C20B—C21B	−88.9 (7)
C16A—C17A—C18A—C19A	−0.1 (14)	Ru2B—As2B—C20B—C21B	39.4 (7)
C17A—C18A—C19A—C14A	0.3 (13)	C13B—As2B—C20B—C25B	−21.0 (8)
C15A—C14A—C19A—C18A	−0.9 (13)	C14B—As2B—C20B—C25B	85.4 (8)
As2A—C14A—C19A—C18A	−172.0 (7)	Ru2B—As2B—C20B—C25B	−146.4 (7)
C14A—As2A—C20A—C25A	−88.8 (7)	C25B—C20B—C21B—C22B	0.2 (13)
C13A—As2A—C20A—C25A	18.2 (8)	As2B—C20B—C21B—C22B	174.8 (7)
Ru2A—As2A—C20A—C25A	143.3 (6)	C20B—C21B—C22B—C23B	−0.9 (14)
C14A—As2A—C20A—C21A	85.1 (7)	C21B—C22B—C23B—C24B	0.9 (14)
C13A—As2A—C20A—C21A	−168.0 (6)	C22B—C23B—C24B—C25B	−0.3 (14)
Ru2A—As2A—C20A—C21A	−42.8 (7)	C23B—C24B—C25B—C20B	−0.4 (14)
C25A—C20A—C21A—C22A	−0.2 (13)	C21B—C20B—C25B—C24B	0.4 (13)
As2A—C20A—C21A—C22A	−174.1 (7)	As2B—C20B—C25B—C24B	−173.9 (7)
C20A—C21A—C22A—C23A	−0.4 (13)	P1B—O10B—C26B—C27B	−113.5 (9)
C21A—C22A—C23A—C24A	0.8 (14)	P1B—O10B—C26B—C31B	69.0 (10)
C22A—C23A—C24A—C25A	−0.6 (14)	O10B—C26B—C27B—C28B	−176.5 (9)
C21A—C20A—C25A—C24A	0.3 (13)	C31B—C26B—C27B—C28B	1.1 (15)
As2A—C20A—C25A—C24A	173.8 (7)	C26B—C27B—C28B—C29B	−0.3 (17)
C23A—C24A—C25A—C20A	0.1 (14)	C27B—C28B—C29B—C30B	−0.4 (17)
P1A—O10A—C26A—C27A	152.0 (8)	C28B—C29B—C30B—C31B	0.2 (16)
P1A—O10A—C26A—C31A	−29.4 (14)	C29B—C30B—C31B—C26B	0.7 (14)
C31A—C26A—C27A—C28A	−0.6 (17)	C27B—C26B—C31B—C30B	−1.3 (14)
O10A—C26A—C27A—C28A	178.0 (10)	O10B—C26B—C31B—C30B	176.1 (8)
C26A—C27A—C28A—C29A	−0.4 (17)	P1B—O11B—C32B—C37B	−106.0 (10)
C27A—C28A—C29A—C30A	1.5 (16)	P1B—O11B—C32B—C33B	76.9 (9)
C28A—C29A—C30A—C31A	−1.7 (16)	C37B—C32B—C33B—C34B	−0.2 (13)
C29A—C30A—C31A—C26A	0.7 (15)	O11B—C32B—C33B—C34B	176.9 (7)
C27A—C26A—C31A—C30A	0.4 (16)	C32B—C33B—C34B—C35B	−0.9 (13)
O10A—C26A—C31A—C30A	−178.0 (9)	C33B—C34B—C35B—C36B	0.8 (14)
P1A—O11A—C32A—C37A	79.0 (11)	C34B—C35B—C36B—C37B	0.4 (15)
P1A—O11A—C32A—C33A	−101.5 (10)	C33B—C32B—C37B—C36B	1.4 (15)
C37A—C32A—C33A—C34A	1.8 (16)	O11B—C32B—C37B—C36B	−175.7 (9)
O11A—C32A—C33A—C34A	−177.7 (9)	C35B—C36B—C37B—C32B	−1.5 (16)
C32A—C33A—C34A—C35A	−1.9 (17)	C38C—O12B—C38B—C39B	99 (8)
C33A—C34A—C35A—C36A	2.0 (19)	P1B—O12B—C38B—C39B	80 (2)
C34A—C35A—C36A—C37A	−2(2)	C38C—O12B—C38B—C43B	−82 (7)
C33A—C32A—C37A—C36A	−1.7 (17)	P1B—O12B—C38B—C43B	−100 (2)
O11A—C32A—C37A—C36A	177.8 (10)	O12B—C38B—C39B—C40B	−177 (2)
C35A—C36A—C37A—C32A	1.8 (19)	C43B—C38B—C39B—C40B	3(3)
P1A—O12A—C38A—C43A	−134.9 (9)	C38B—C39B—C40B—C41B	−3(4)
P1A—O12A—C38A—C39A	50.9 (13)	C39B—C40B—C41B—C42B	−7(4)
C43A—C38A—C39A—C40A	−1.3 (15)	C40B—C41B—C42B—C43B	16 (4)
O12A—C38A—C39A—C40A	172.6 (9)	C41B—C42B—C43B—C38B	−15 (4)
C38A—C39A—C40A—C41A	3.8 (15)	O12B—C38B—C43B—C42B	−174 (2)
C39A—C40A—C41A—C42A	−2.1 (16)	C39B—C38B—C43B—C42B	6(4)
C40A—C41A—C42A—C43A	−2.0 (16)	C38B—O12B—C38C—C43C	103 (8)
O12A—C38A—C43A—C42A	−177.3 (9)	P1B—O12B—C38C—C43C	−93 (3)
C39A—C38A—C43A—C42A	−2.9 (15)	C38B—O12B—C38C—C39C	−79 (7)
C41A—C42A—C43A—C38A	4.5 (15)	P1B—O12B—C38C—C39C	85 (3)
C45B—Ru1B—Ru2B—C48B	19.1 (17)	C43C—C38C—C39C—C40C	−19 (5)
C46B—Ru1B—Ru2B—C48B	−60.0 (11)	O12B—C38C—C39C—C40C	163 (3)
C44B—Ru1B—Ru2B—C48B	119.1 (11)	C38C—C39C—C40C—C41C	23 (6)
As1B—Ru1B—Ru2B—C48B	−155.1 (11)	C39C—C40C—C41C—C42C	−19 (6)
Ru3B—Ru1B—Ru2B—C48B	21.7 (11)	C40C—C41C—C42C—C43C	10 (4)
C45B—Ru1B—Ru2B—C47B	97.6 (13)	C41C—C42C—C43C—C38C	−6(4)
C46B—Ru1B—Ru2B—C47B	18.4 (4)	C39C—C38C—C43C—C42C	10 (5)
C44B—Ru1B—Ru2B—C47B	−162.5 (4)	O12B—C38C—C43C—C42C	−172 (3)
As1B—Ru1B—Ru2B—C47B	−76.7 (3)		

### Hydrogen-bond geometry (Å, °)

**Table:**

Cg1, Cg2, Cg3 , Cg4, Cg5, Cg6 and Cg7 are the centroids of the C1B–C6B, C1A–C6A, C14A–C19A, C14A–C19A, C7A–C12A, C20B–C25B and C20A–C25A benzene rings, respectively.

**Table:**

*D*—H···*A*	*D*—H	H···*A*	*D*···*A*	*D*—H···*A*
C31A—H31A···O12A	0.93	2.42	3.014 (13)	121
C39A—H39A···O11A	0.93	2.38	2.959 (11)	120
C43B—H43B···O2B	0.93	2.53	3.42 (2)	158
C11A—H11A···Cg1^i^	0.93	2.95	3.742 (12)	144
C11B—H11B···Cg2^ii^	0.93	2.85	3.664 (10)	147
C16A—H16A···Cg1^iii^	0.93	2.82	3.668 (10)	151
C16B—H16B···Cg2	0.93	2.82	3.627 (10)	146
C24A—H24A···Cg3^iv^	0.93	2.96	3.719 (10)	140
C24B—H24B···Cg4^v^	0.93	2.91	3.679 (10)	141
C36B—H36B···Cg5^ii^	0.93	2.85	3.756 (12)	166
C40A—H40A···Cg6^vi^	0.93	2.82	3.660 (10)	150
C40B—H40B···Cg7^vii^	0.93	2.71	3.55 (2)	150
C40C—H40C···Cg7^vii^	0.93	2.70	3.56 (3)	154

Symmetry codes: (i) −*x*+1, *y*+1/2, −*z*+3/2; (ii) −*x*, *y*−1/2, −*z*+3/2; (iii) *x*, *y*+1, *z*;  (iv) −*x*, *y*+1/2, −*z*+3/2; (v) −*x*+1, *y*−1/2, −*z*+3/2; (vi) −*x*+1, −*y*+1, −*z*+1; (vii) −*x*, −*y*+1, −*z*+1.
